# The mechanism of electrical remodeling in atrial fibrillation and current research status of natural drugs and active ingredients inhibiting atrial electrical remodeling

**DOI:** 10.3389/fcvm.2026.1705565

**Published:** 2026-03-11

**Authors:** Xiangyuan Huang, Ci Wang, Yanyun Wang, Shuyu Yang, Linshan Du, Liangzhi Li, Tianyi Wang, Ying Lu

**Affiliations:** 1 Beijing University of Chinese Medicine, Beijing, China; 2 The First Affiliated Hospital of Zhejiang Chinese Medical University, Hangzhou, Zhejiang, China; 3 Tianjin University of Traditional Chinese Medicine, Tianjin, China

**Keywords:** cardiac electrophysiology, active ingredients, atrial fibrillation, electrical remodeling, natural drug

## Abstract

Atrial fibrillation (AF) is the most common arrhythmia in clinical practice, with its incidence and mortality rates steadily increasing every year, posing a growing global health threat. During AF, high-frequency sustained electrical activity in atria induces electrophysiological changes. This process, known as atrial electrical remodeling (AER), in turn, plays a crucial role in both the initiation and maintenance of AF. Key contributors to AER include abnormalities in gene expression, alterations in electrophysiology, dysfunction of the ion channel, inflammatory responses, and oxidative stress. Natural compounds, primarily derived from plants and other natural resources, have attracted considerable attention for their high efficacy, low toxicity, and ability to target multiple therapeutic pathways. These compounds—such as matrine, berberine, ginsenosides, quercetin, icariin, and tanshinone—offer comprehensive regulatory effects that can effectively attenuate AER, providing unique therapeutic advantages in the management of AF. This review systematically synthesizes the mechanisms underlying AER and examines how natural drugs and their active ingredients can mitigate AF by inhibiting this remodeling process, offering valuable insights for the development of potentially effective natural therapeutics against AF.

## Graphical Abstract

**Figure:**
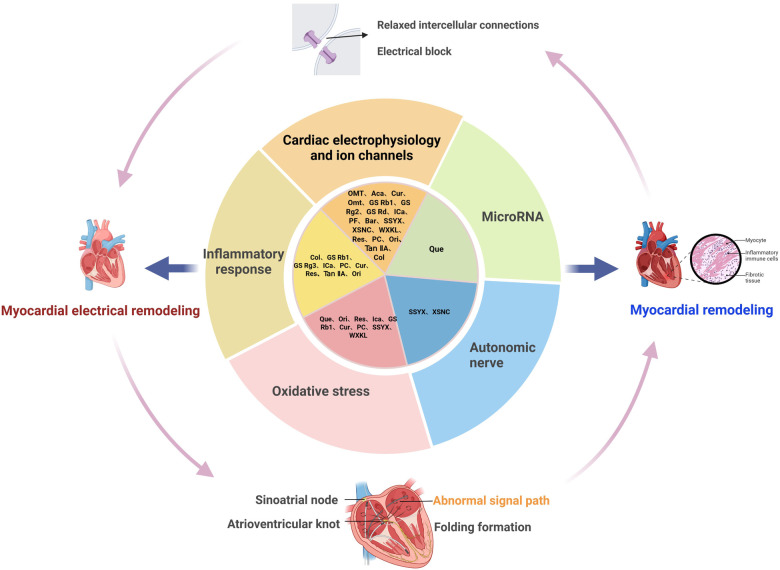

## Highlights

- Revealing the complex mechanisms of atrial electrical remodeling (AER) in atrial fibrillation (AF) profoundly.
- Systematically classifying and reviewing the active components of natural medicines targeting AER, and elucidate their mechanisms of action.
- Proposing valuable therapeutic perspectives tailored to the mechanism of AER in AF.

## 1 Introduction

Atrial fibrillation (AF) is the most common cardiac arrhythmia encountered in clinical practice. During AF episodes, the atria fail to either generate or replace the normal electrical signals from the sinoatrial node, leading to diffuse and disorganized electrical activity. This disruption results in the deterioration or complete loss of atrial pumping function. Clinically, AF primarily manifests as palpitations, shortness of breath, syncope, fatigue, etc., which significantly impact the quality of life of patients and is one of the most important factors contributing to disability and mortality (1). The presence of AF increases the risk of stroke by fivefold and death by 40%–90% (2). As of 2010, approximately 33.5 million individuals worldwide were living with AF, and both its prevalence and incidence are rising with age across various regions (3). A longitudinal health and longevity survey conducted in China through 2012 revealed an overall AF prevalence rate of 3.5% among individuals over 65 years of age, with the rate increasing to 8.8% among those aged 80 and older (4). With the growing global aging population, the prevalence of AF has risen significantly, positioning AF as a major and growing public health concern. As of 2023, approximately 60 million individuals worldwide were affected by AF (5). Projections suggest that by 2050, more than 10 million people in the United States will be affected by AF, while by 2060, the number of affected individuals in Europe is expected to reach 17.9 million (6, 7).

The mechanisms underlying the onset and progression of AF are broadly classified into electrophysiological and pathophysiological mechanisms. Electrophysiological hypotheses include focal trigger mechanisms, multiple wavelet reentry, focal excitation, and the rotor theory, among others (8, 9). Pathophysiological mechanisms encompass genetic predispositions, atrial remodeling, autonomic nervous system (ANS) remodeling, inflammatory responses, and oxidative stress. Atrial remodeling, which comprises both electrical and structural changes, is a critical substrate for the initiation and perpetuation of AF. Atrial electrical remodeling (AER) is ultimately attributed to changes in the function or expression of ion channels in atrial myocytes (10, 11). Its main electrophysiological characteristics include the shortening of the atrial effective refractory period (AERP) and action potential duration (APD), slowing of action potential conduction velocity, and enhanced variability in the refractory period. AER leads to the generation of discrete electrical signals and multiple reentrant waves within atria, which are crucial in driving the onset and maintenance of AF.

The treatment of AF primarily includes pharmacological strategies—such as anticoagulation, ventricular rate control, and cardioversion—as well as non-pharmacological interventions such as radiofrequency ablation and the implantation of implantable cardioverter-defibrillators (12). However, antiarrhythmic drugs often demonstrate limited long-term efficacy and are associated with considerable cardiac neurotoxicity and extracardiac toxicity, resulting in notable side effects and adverse reactions. Class I antiarrhythmic agents, such as flecainide, may worsen cardiac function and increase mortality in patients with ischemic heart disease or structural heart abnormalities (13). Similarly, Class III agents, such as amiodarone, are known to prolong the QT interval and induce a spectrum of extracardiac adverse effects (14). Despite their therapeutic potential, non-pharmacological treatments often encounter limited patient acceptance because of high costs and an elevated risk of postprocedural recurrence.

Natural drugs and their active ingredients, primarily derived from plants and other natural sources, possess characteristics such as high efficacy, low toxicity, and the ability to target multiple pathways and receptors. Through synergistic or antagonistic actions within complex biological networks, these agents can mitigate the side effects typical of single-pathway drugs. This multitarget approach offers distinct clinical advantages and therapeutic potential (15). By targeting and modulating AER, such natural substances may effectively inhibit the onset and progression of AF. Accordingly, a comprehensive review of the mechanisms underlying AER in AF, alongside an evaluation of current research on natural agents targeting this remodeling process, is of significant scientific and clinical value.

## 2 Electrophysiological mechanisms of AF

AER involves alterations in the electrophysiological properties of atrial myocardium, primarily resulting from changes in atrial myocyte ion channels induced by recurrent episodes of AF (16). AER may be triggered by various factors, including dysregulated gene expression, ANS remodeling, inflammatory responses, and oxidative stress. These alterations collectively disrupt cardiac electrophysiology and ion channel function, potentially promoting the progression of paroxysmal AF to persistent or even permanent AF. In addition, the heart undergoes a series of pathological adaptations in response to AF. Although some of these adaptations may initially serve compensatory purposes, they can also develop into novel arrhythmogenic substrates, further facilitating the initiation and maintenance of AF (17).

### 2.1 Ion channel alterations and electrophysiological changes in AER

The functional activity of cardiac ion channels and electrophysiological changes are closely associated with AER. Alterations in ion channel expression and function form the fundamental basis of AER. During the initiation of AF, ion efflux increases, primarily through ATP-sensitive potassium currents (I_K−ATP_) and inward rectifier potassium currents (I_K1_). Simultaneously, L-type calcium currents (I_Ca, L_) and transient outward potassium currents (I_to_) are reduced, resulting in an accelerated repolarization phase (18). These ionic changes cause electrophysiological alterations manifested primarily as shortening of the AERP and APD. Rapid and sustained atrial stimulation or recurrent AF episodes progressively establish a self-perpetuating state of AF (19).

**Figure 1:**
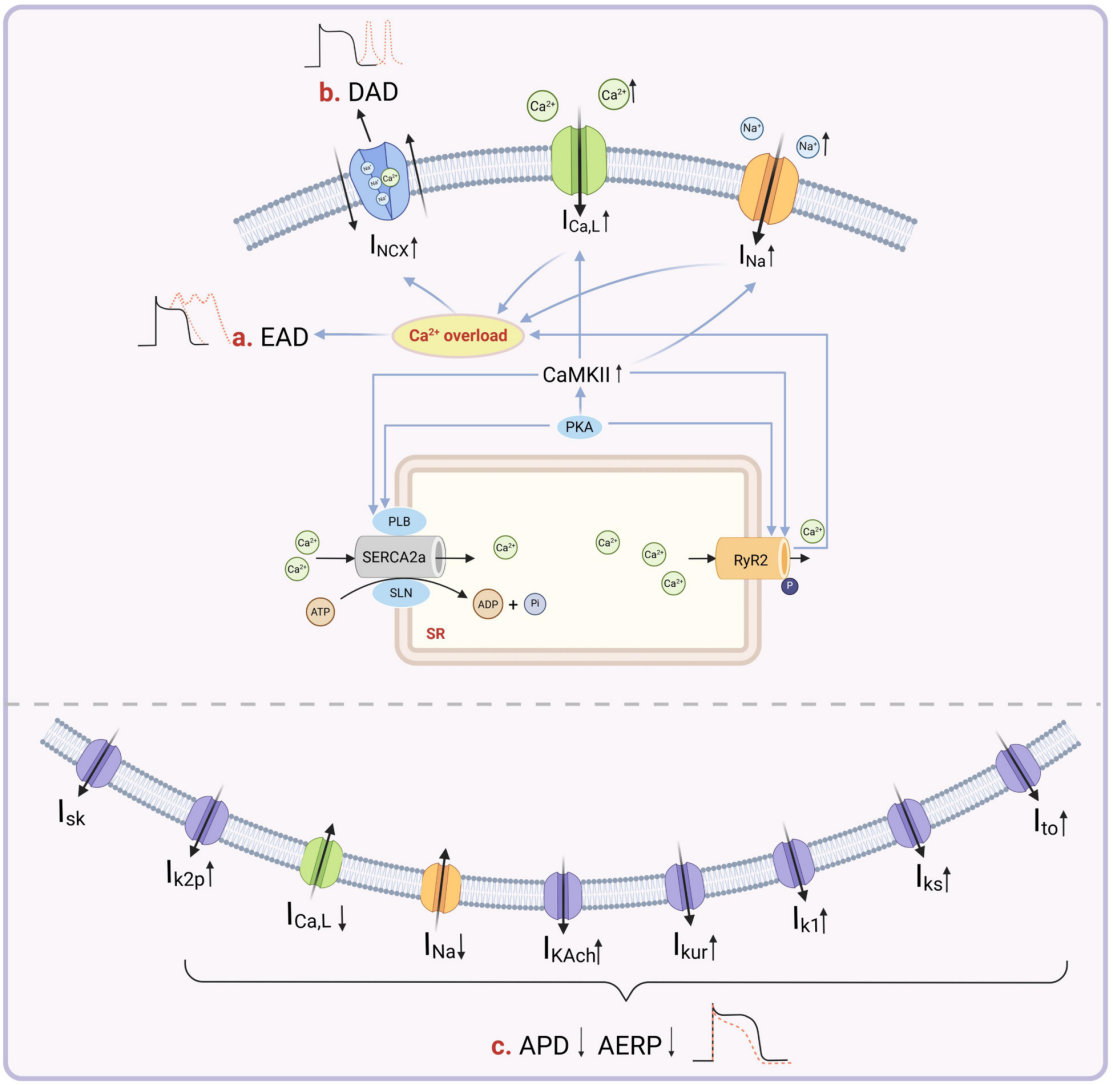
Ion channels and electrophysiological alterations in myocardial cells related to atrial electrical remodeling. **(a)** The upregulation of CaMKII results in an increased I_Na_, leading to sodium-dependent calcium overload. This condition, along with other contributing factors, culminates in a comprehensive calcium overload that triggers AF by inducing EAD. **(b)** The hyperphosphorylation of RyR2, mediated by CaMKII, causes an abnormal release of SRCa^2+^. This release, combined with an increased influx of Ca^2+^, results in an overload of calcium within the cell. Subsequently, this activates the NCX, which enhances the expulsion of calcium from the cell, leading to delayed DAD and sustaining AF. **(c)** Reductions in I_Ca,L_ and I_Na_, along with increases in I_K−ATP_, I_Ks_, I_Kur_, I_K1_, and I_K2p_, contribute to the shortening of the APD and AERP, thus facilitating atrial electrical remodeling. By Figdraw (http://www.figdraw.com).

The onset of AF is accompanied by a slowing of sodium channel inactivation (20). During the rapid inactivation phase of sodium channels, a small fraction does not remain inactivated. Instead, these channels either reopen or stay open during the repolarization phase of the action potential, called the late sodium current (I_Na−L_) (21). In the initial rapid repolarization phase, I_to_ flows out quickly, causing a rapid and brief drop in the membrane's internal potential. Phase 2 of repolarization is relatively prolonged. It involves multiple ion currents, including inward currents such as I_Ca, L_ and I_Na−L_, and outward currents like the delayed rectifier potassium current (I_K_). The depolarizing effect of the inward currents is counterbalanced by the repolarizing effect of the outward currents, jointly forming a “plateau phase” (22). During the onset of AF, there is a marked upregulation in the expression of channel proteins associated with the slow delayed rectifier potassium current (I_Ks_), rapid delayed rectifier potassium current (I_Kr_), and ultrarapid delayed rectifier potassium current (I_Kur_). This results in an increased density of the I_K_, leading to a heightened outflow of potassium (K^+^) (23). Although the I_Na−L_ is somewhat enhanced during this phase, the density of the I_Ca, L_ is significantly reduced. The reduction in I_Ca, L_ is a primary factor contributing to the shortening of the AERP in AF (24). As the slow inactivation of I_Ca, L_, and I_Na−L_ progresses and the outward potassium currents strengthen, the membrane's internal potential rapidly declines, leading to the formation of the rapid repolarization phase, phase 3. The inward rectifier phenomenon diminishes, and I_K−ATP_ and I_K1_ are predominantly expressed in the later stages of phase 3. During AF, rapid excitation of the atrial myocytes increases energy expenditure and leads to ischemia and hypoxia, thereby activating the opening of ATP-sensitive potassium channels (K_ATP_). This increase in K^+^ outflow accelerates repolarization and shortens the AERP (25). In patients with chronic AF, the I_K1_ outward current is also significantly enhanced in atrial myocytes. This can lead to hyperpolarization of the atrial muscle cell membrane, further promoting the shortening of the APD and increasing the atrial rate. At the end of phase 3, the sodium-potassium ATPase (Na^+^-K^+^-ATPase/NKA) on the cell membrane is activated, leading to the expulsion of previously influxed Na^+^ and calcium (Ca^2+^). As a result, the ionic composition of the cardiac muscle cells gradually returns to normal, culminating in phase 4, the resting phase. In AF, the expression of acetylcholine-activated inwardly rectifying potassium current (I_KACh_) channel proteins is upregulated. This increases the membrane's permeability to K^+^, enhancing the outflow of K^+^ and making the cell membrane more prone to hyperpolarization, thereby shortening the APD (Figure 1) (26).

#### 2.1.1 Ion channel changes and AER

##### 2.1.1.1 Calcium channel

Dysregulation of intracellular Ca^2+^ homeostasis is implicated in a variety of pathological conditions (27). Calcium channels are broadly categorized into calcium entry channels and calcium release channels. Entry channels include voltage-dependent calcium channels (VDCCs) and ligand-gated calcium channels (LGCCs). Calcium release channels include the ryanodine receptor (RyR) channel and the inositol 1,4,5-triphosphate receptor (IP3R) channel. The VDCCs in cardiomyocytes mainly include L-type calcium channels (LTCCs) and T-type calcium channels (TTCCs). Among these, the LTCC plays a crucial role in mediating changes in action potentials (28).

Disruption of intracellular Ca^2+^ homeostasis, particularly Ca^2+^ overload and delayed after depolarization (DAD), which are caused by dysfunction of key factors such as the Na^+^-Ca^2+^ exchanger (NCX), ryanodine receptor 2 (RyR2), and sarco/endoplasmic reticulum Ca^2+^-ATPase (SERCA), represents a critical component of AER in AF. An increase in the inward sodium current (I_Na_) impairs NCX function, thereby Ca^2+^ efflux, and causes secondary intracellular Ca^2+^ overload. Additional factors, including aberrant gene expression, elevated levels of reactive oxygen species (ROS), and inflammatory responses, further destabilize ion channel function, either directly or indirectly promoting Ca^2+^ overload (29). SERCA2a, an ATP-driven Ca^2+^ pump, plays a vital role in regulating myocardial contractility. It transports two cytosolic Ca^2+^ ions into the sarcoplasmic reticulum (SR) per ATP molecule hydrolyzed. This activity is essential for maintaining cytoplasmic Ca^2+^ concentration, regulating SR Ca^2+^ load, and fine-tuning cardiac contraction and relaxation rates. Disruption of Ca^2+^ homeostasis activates Ca^2+^/calmodulin-dependent protein kinase II (CaMKII), which mediates hyperphosphorylation of RyR2, leading to enhanced SR Ca^2+^ leakage. Dysregulation of calcium homeostasis activates CaMKII, which mediates excessive phosphorylation of RyR2, leading to enhanced calcium leakage from the sarcoplasmic reticulum (30). This abnormal SR Ca^2+^ release into the VDCC activates excitation–contraction coupling in atrial myocytes, intensifying NCX function, promoting further SR Ca^2+^ release, and resulting in spontaneous SR Ca^2+^ release and DAD. The Ca^2+^-induced Ca^2+^ release mechanism plays a pivotal role in AER (31, 32). Alterations in the LTCC are also closely associated with AER. Downregulation of LTCC gene expression reduces I_Ca, L_ density, which shortens the APD and AERP, thereby facilitating reentrant excitation and increasing susceptibility to AF (33).

##### 2.1.1.2 Potassium channel

Potassium channels are crucial for myocardial repolarization. They are key regulators of the resting membrane potential and APD. Potassium currents are categorized into two major types in human atrial myocytes: voltage-dependent and receptor-activated potassium currents. Voltage-dependent currents include I_to_, I_K_, and I_K1_. In contrast, receptor-initiated currents mainly comprise I_K−ATP_ and I_KACh_ (34).

Voltage-gated potassium channels encompass multiple subfamilies, with I_to_ dominating the early repolarization phase of cardiomyocytes. The I_K_, including components such as I_Ks_, I_Kr_, and I_Kur_, jointly regulate the repolarization process, transitioning the action potential from the plateau phase back to the resting potential (35). In the heart, the inwardly rectifying potassium channel subfamilies Kir2.x, Kir3.x, and Kir6.x encode the I_K1_, I_KACh_, and K_ATP_ channels, respectively (36). I_K1_ plays a vital role during phase 3 repolarization and is crucial for sustaining the resting membrane potential. The K_ATP_ channel is an inwardly rectifying current activated by ATP depletion. It helps stabilize the resting membrane potential and aids in phase three repolarization (37).

Dysfunction of potassium channels is closely associated with the development of AER. An abnormal increase in I_Kur_ is an undesirable factor in the triggering of AF. Blocking I_Kur_ current prolongs the action potential plateau, which, in turn, increases the APD and AERP. Consequently, the cardiac calcium transient (CaT) is amplified, producing a positive inotropic effect (38, 39). Similarly, an increase in I_Ks_ contributes to the shortening of the AERP and APD in atrial myocytes, thereby perpetuating AF by promoting the conduction of protofibers (40). I_KACh_ plays a critical role in stabilizing reentrant circuits during AF. The initiation and maintenance of AF are significantly influenced by the activity of acetylcholine-activated inwardly rectifying potassium channel (K_ACh_). Accordingly, pharmacological inhibition of K_ACh_ represents a promising therapeutic strategy for treating AF (41). In studies involving the infusion of the I_KACh_ inhibitor XAF-1407, a dose-dependent prolongation of AERP was observed, indicating a distinct atrial-selective effect. This suggests a dose-dependent inhibitory impact of I_KACh_ on the AERP (42). In addition, the upregulation of I_K1_ aids in stabilizing the atrial rotor, leading to a reduced APD and playing a role in both the stabilization and maintenance of AF (43).

Recent studies reveal that the two-pore-domain potassium channel (K_2P_) [such as TASK-1 (K2P3.1)] and the small-conductance calcium-activated potassium channel (SK) play crucial roles in AER. Their functional abnormalities are associated with shortened atrial APD and increased susceptibility to AF, potentially making them promising therapeutic targets for AF treatment (44–46).

SK channels comprise SK1, SK2, and SK3 isoforms, with the SK2 isoform being predominantly located in atrial myocardium. These channels contribute significantly to the repolarization phase of the action potential in atrial myocytes (47). Notably, both hyperactivation and inhibition of SK channels have been shown to increase the susceptibility to AF. Therefore, maintaining optimal expression and functional balance of SK channel proteins may represent a novel and promising strategy for managing AF (48, 49).

##### 2.1.1.3 Sodium channel

Sodium channel currents are the major ion channel currents of phase 0 depolarization in fast-responding cardiomyocytes and are responsible for the initiation and propagation of action potentials in the nerve, muscle, and heart. Sodium currents include I_Na_ and I_Na−L_. Pathologic increases in I_Na−L_ are associated with arrhythmic phenotypes due to inherited heart disease and acquired heart disease such as long QT syndrome (LQT), AF, and myocardial infarction. The remodeling of I_Na−L_ differs between paroxysmal and persistent AF (50). In the ΔKPQ mouse model of LQT3, elevated I_Na−L_ causes an extended APD in atrial tissue, highlighting its significance in treating atrial arrhythmias in patients with LQTS by inducing early after depolarization (EAD) (51).

The voltage-gated sodium channel Na_V_1.5 plays a crucial role in initiating and propagating cardiac action potentials. Genetic variations in SCN5A, the primary coding gene for Na_V_1.5, are known to shorten the APD, alter cardiac repolarization, and increase the risk of AF (52). Calcium channels have a close association with sodium channels. In the CaMKⅡ-transgenic mouse model, overexpression of CaMKⅡ*δ*C enhances I_Na−L_, thereby prolonging the APD and inducing EAD or LQT3-like arrhythmic events. The phosphorylation of Na_V_1.5 by CaMKⅡ is a significant arrhythmogenic mechanism, regulating sodium channel function and contributing to arrhythmia susceptibility (53, 54).

#### 2.1.2 Electrophysiological changes and AER

##### 2.1.2.1 AERP

The AERP spans the repolarization period from phase 0 depolarization until the membrane potential recovers to approximately −60 mV. During this period, regardless of intensity, the cell does not respond to further stimulation. AER is characterized by a marked shortening of the AERP, primarily resulting from decreased I_Ca, L,_ and increased I_K1_. These ionic changes increase the susceptibility of atrial myocytes to aberrant electrical activity and impair the termination of ectopic impulses. This promotes the formation of extensive areas with heterogeneous functional refractoriness. The degree of AERP shortening progressively worsens with the duration of AF. Electrical remodeling not only reduces the absolute AERP duration but also disrupts its rate dependence and spatial distribution. The adaptive loss or reversal of normal AERP rate dependence increases the heterogeneity and dispersion of electrical conduction across the atria. This promotes the formation of reentrant circuits. These developments contribute significantly to the persistence and progression of AF (55–57).

##### 2.1.2.2 APD

The APD encompasses the interval from the onset of cardiomyocyte depolarization to the completion of repolarization (58). The length of the APD influences cardiac contraction and relaxation as well as cardiac rhythm stability (59). Shortening of the APD and increased APD variability are crucial for initiating and sustaining reentrant circuits, thereby driving AF development (60). A shortened APD enhances the vulnerability and persistence of AF. Factors contributing to the shortening of the APD include Ca^2+^ overload, which subsequently leads to changes such as a reduction in I_Ca, L_, and an increase in I_K1_. In addition, APD shortening is influenced by various other factors, including miRNA regulation, alterations in ion channel expression, inflammatory responses, and oxidative stress (61, 62).

### 2.2 MiRNA and AER

**Figure 2:**
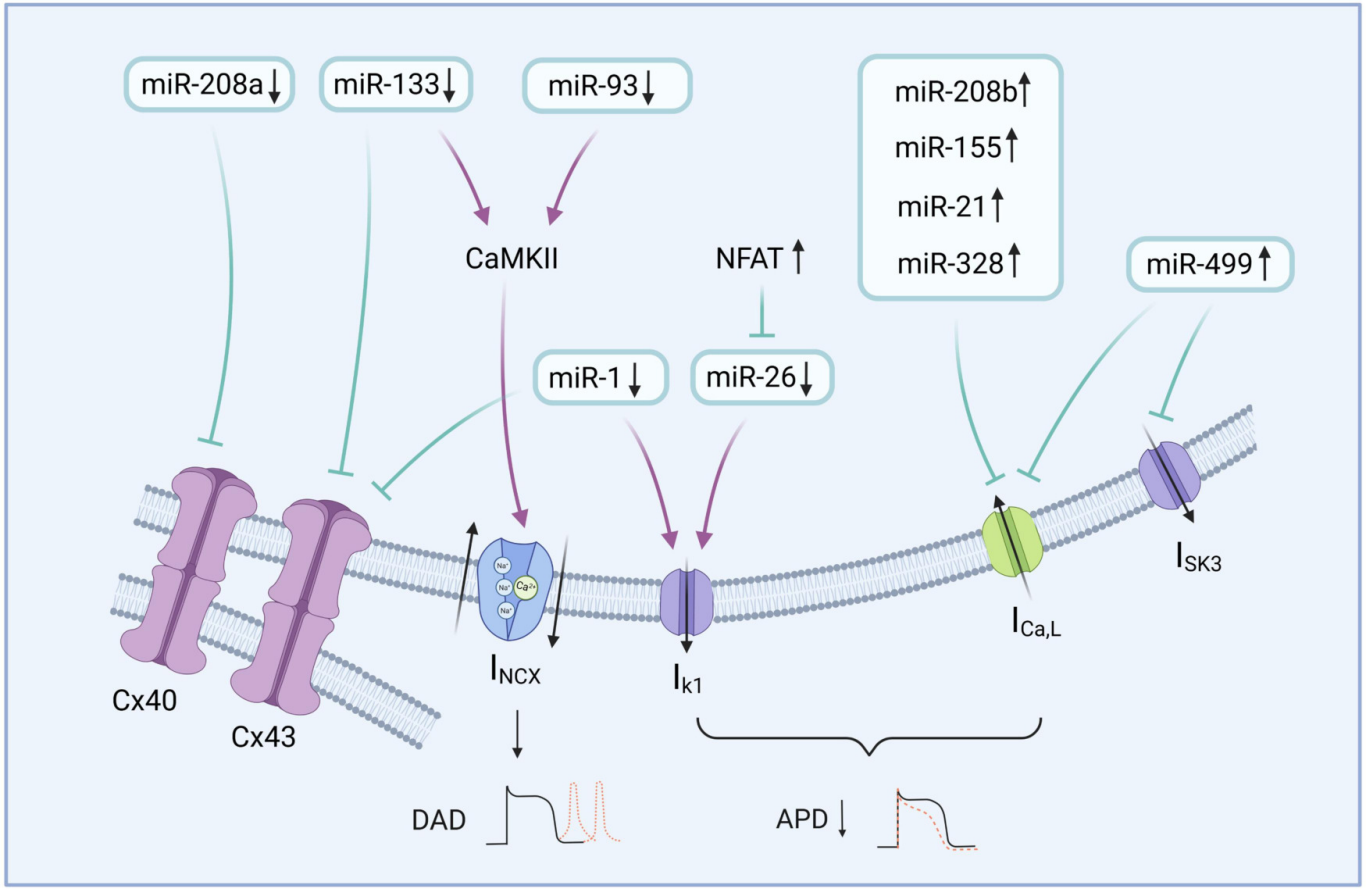
miRNA and atrial electrical remodeling. miRNA plays a crucial role in the regulation of gene expression and is involved in multiple regulatory processes of atrial electrical remodeling. By Figdraw (http://www.figdraw.com).

MiRNAs regulate gene expression by binding to the 3′-untranslated region (3′-UTR) of target mRNAs, leading to their degradation or translational inhibition (63). As key molecules regulating gene expression, miRNAs serve as important biomarkers and potential therapeutic targets for various cardiovascular diseases, including AF (64). Growing evidence indicates that miRNAs are involved in the regulation of AER. Different miRNAs play different roles in AF depending on the underlying etiology of AF, which is important for the development of potential drug targets for the treatment of AF (Figure 2) (65).

#### 2.2.1 MiR-328

MiR-328 levels are significantly elevated in patients with AF (66). Upregulation of miR-328 has been shown to markedly increase AF susceptibility and prolong its duration. This mechanism may be related to the reduction of I_Ca, L_ levels and the shortening of atrial APD. Further studies have confirmed that the genes calcium voltage-gated channel subunit alpha 1c (CACNA1C) and calcium voltage-gated channel auxiliary subunit beta (CACNB1), which encode the α1c and β1 subunits of the LTCC, are downstream targets of miR-328 regulation (66). However, the mechanisms underlying the autocrine and paracrine actions of miR-328 remain poorly understood, and further research is needed to elucidate their roles in atrial remodeling (67).

#### 2.2.2 MiR-133

MiR-133 plays a crucial role in AER. Functional studies involving miR-133 overexpression, targeted deletion, and antisense knockdown have identified its regulatory roles and molecular targets in cardiac remodeling (68, 69). As_2_O_3_-induced elevation of miR-133 levels prolongs the QT interval and enhances cardiac toxicity (70). Another study using miR-133 mimics demonstrated that miR-133 suppresses zinc finger home obox3-knock down (ZFHX3-KD)-mediated cardiac remodeling signaling pathways. This mechanism involves downregulating the activity of the dishevelled 2/CaMKⅡ/c-Jun N-terminal kinase (DVL2/CaMKⅡ/JNK) signaling pathway and reducing the SR Ca^2+^ content. It also suppresses the expression of CACNB1 and protein kinase, thereby effectively inhibiting the progression of AF. Upregulation of miR-133 levels may be a potential therapeutic approach for AF patients with ZFHX3 dysfunction (69).

#### 2.2.3 MiR-1

MiR-1 is the most abundantly expressed miRNA in the myocardium and its expression is downregulated in patients with AF (71–73). In models of miR-1 overexpression, expression levels of the inward rectifier potassium channel Kir2.1 and gap junction protein connexin 43 (Cx43) are significantly reduced, leading to shortened AERP and enhanced AF susceptibility through a decrease in the IK_1_. Knockdown of miR-1 targets potassium channel subunits KCNE1 and KCNB2, thereby reversing AERP shortening and mitigating AF vulnerability by modulating potassium channel remodeling (74).

#### 2.2.4 MiR-26

MiR-26 is a vertebrate-specific microRNA whose expression is upregulated under hypoxic conditions and is closely associated with cellular growth and development (75). Downregulation, inhibition, or mutation of the miR-26 binding site has been shown to promote both the initiation and maintenance of AF. During AF, activation of the nuclear factor of activated T cells (NFAT) pathway suppresses the expression of miR-26 and its downstream target gene *KCNJ2*, leading to upregulation of the IK_1_ and shortening of the atrial APD, thereby facilitating AF persistence. The administration of miR-26 via adenoviral gene delivery through the tail vein effectively inhibited the onset and maintenance of AF, indicating a promising therapeutic potential for miR-26 in the treatment of AF (76).

#### 2.2.5 MiR-21

MiR-21 is one of the earliest identified mammalian miRNAs. Its expression is markedly upregulated during pathological conditions such as inflammation and myocardial fibrosis (77). In patients with AF, miR-21 is significantly overexpressed, with its average expression level in the left atrial tissue reported to be approximately 2.5-fold higher than that in patients maintaining sinus rhythm (78). MiR-21 downregulates Ca2+ channel protein expression through direct binding to the 3′-UTR of LTCC subunits (CACNA1C and CACNB2), which, in turn, reduces I_Ca,L_ density and is involved in AER (79).

#### 2.2.6 MiR-499

Upregulated miR-499 promotes AER by simultaneously affecting calcium and potassium channels. Specifically, miR-499 downregulates the expression of the voltage-dependent calcium channel β-2 subunit (CACNB2) by inhibiting its translation process, thereby promoting AER (80). In addition, miR-499 can directly bind to the mRNA encoding the SK3 gene, leading to a reduction in SK3 expression and inducing AF (81).

#### 2.2.7 MiR-155

MiR-155 plays a key role in various physiological and pathological processes, including cardiovascular diseases (82). Downregulation of endogenous miR-155 has been shown to exert a protective effect against AF. In miR-155 knockout hearts, AER was attenuated, which is at variance with the results in the miR-155 overexpression mouse model. The mechanism of miR-155 causing AF may be related to the reduction of I_Ca, L_, and shortening of APD (83).

#### 2.2.8 MiR-93

MiR-93 belongs to the miR-17 cluster (84). As a member of the miR-106b-25 cluster, miR-93 is a major regulator involved in RyR2-mediated SR Ca^2+^ release. It can downregulate RyR2 expression to reduce AF susceptibility (85). Impaired miRNA-mediated dysregulation of RyR2 increases AF susceptibility in mice. Aberrant Ca^2+^ leakage from RyR2 may be a source of ectopic activity in various AF models, which sheds light on the study of the pathogenesis of paroxysmal AF in humans (86).

#### 2.2.9 MiR-208

The miR-208 family is cardiac-specific and mainly consists of miR-208a and miR-208b. MiR-208a, one of the most highly expressed heart-enriched microRNAs, participates in multiple cardiac pathological processes such as myocardial fibrosis, hypertrophy, and heart failure (87, 88). MiR-208a serves as a biomarker for AF severity, exhibiting moderate expression in paroxysmal AF, increased levels in persistent AF, and significantly decreased expression in long-term persistent AF (*p* = 0.02) (89). Connexin 40 (Cx40) is the major connexin in atrial myocardium. MiR-208a-3p acts as a critical upstream negative regulator of Cx40 in patients with chronic AF. It may contribute to AF pathogenesis by indirectly promoting Cx40 remodeling. Consequently, downregulation of miR-208a-3p presents a potential therapeutic target for AF (90, 91).

MiR-208b expression is elevated in chronic AF. Overexpression of miR-208b inhibits the expression and function of LTCC subunits, including CACNA1C and CACNB2, resulting in decreased SR Ca^2+^ loading and release and indirectly altering the distribution of Cx43 in cell gap junctions (92). Further studies indicate that miR-208b mimics directly bind to the 3′-untranslated regions (3′-UTRs) of CACNA1C and KCNJ5. This finding suggests that miR-208b, specifically upregulated in AF, may serve as a key mediator of abnormal Ca^2+^ handling during atrial remodeling (93).

### 2.3 Inflammatory response and AER

**Figure 3:**
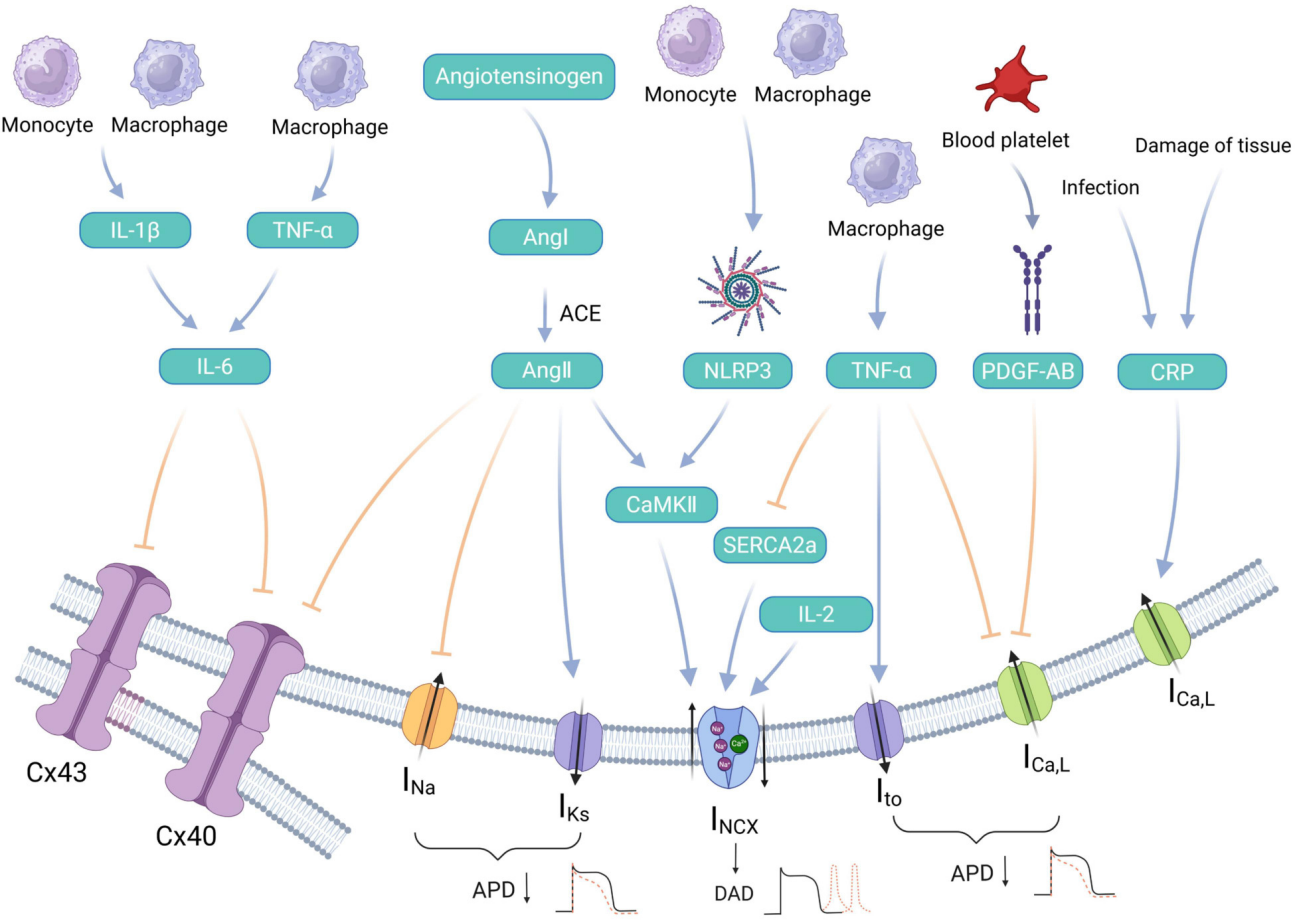
Inflammatory responses and atrial electrical remodeling. Inflammatory factors that are involved in affecting atrial electrical remodeling primarily include PDGF, Ang Ⅱ, NLRP3, TNF-α, IL, and CRP. Among them, IL-6 and Ang Ⅱ slow down atrial conduction speed by inhibiting Cx, triggering an imbalance in local myocardial electrical activity. Ang Ⅱ, NLRP3, and TNF-α induce DAD by upregulating NCX protein activity. PDGF-AB, TNF-α, and Ang Ⅱ can jointly mediate the shortening of the APD by acting on sodium, potassium, and calcium channel proteins. CRP can induce calcium overload by upregulating I_Ca,L_. Various inflammatory responses collectively promote the onset and development of atrial fibrillation by affecting atrial electrical remodeling. By Figdraw (http://www.figdraw.com).

The inflammatory response, a physiological reaction to harmful stimuli, facilitates the body's transition to a new state of homeostasis and is intricately linked to the development of AF. In the early stages of AF, leukocyte infiltration increases markedly within atrial tissues. Inflammatory cells such as monocytes, macrophages, and lymphocytes contribute to thrombus formation by producing cytokines and chemokines (94). During the intermediate stage of AF, leukocyte infiltration in atrial tissue remains elevated, and the combined production of tumor necrosis factor-alpha (TNF-α) and interleukins (IL) by inflammatory cells accelerates atrial fibrosis and cardiomyocyte apoptosis. In late-stage AF, the number of inflammatory cells in atrial tissue declines, but their activity persists. This sustained activity continues to exacerbate atrial fibrosis. Accompanied by abnormal expression and functional changes in ion channels, further AER occurs. Inflammatory response mediators and associated pathways primarily affect the LTCC, NCX, and Ca^2+^ pumps, leading to shortened APD and AERP and promoting AER. Conversely, AF intensifies the inflammatory response, creating a clinical cycle where “AF begets AF” (95). This section highlights the roles of several key inflammatory mediators in the electrical remodeling of the atrium, exemplifying their significance (Figure 3).

#### 2.3.1 Platelet-derived growth factor

Platelet-derived growth factor (PDGF) is a cationic protein mainly stored in platelet granules. It can be secreted by various cells, including monocytes/macrophages, endothelial cells, and vascular smooth muscle cells (96). The PDGF family comprises four unique isoforms: PDGF-A, PDGF-B, PDGF-C, and PDGF-D. These isoforms dimerize to form active proteins, producing four homodimers and one heterodimer (PDGF-AB) (97). PDGF contributes to AER by modulating calcium channel function and shortening the APD. PDGF-AB induces mislocalization of the intracellular CaV1.2 protein, which downregulates its expression. This reduces I_Ca, L_ density and shortens the APD, ultimately promoting AF. PDGF-AA isoform dimer induces atrial fibrosis and enhances AF susceptibility (98, 99).

#### 2.3.2 Angiotonin Ⅱ

Angiotonin Ⅱ (Ang Ⅱ) is a major effector of the renin–angiotensin system (RAS), which plays a key role in regulating the physiological processes of the cardiovascular system. Elevated levels of Ang II are closely associated with the development of AER. Inositol 1,4,5-trisphosphate (IP3), acting as a secondary messenger for Ca^2+^, significantly boosts CaT in atrial myocytes. It achieves this by increasing the Ca^2+^ concentration around RyRs, thereby regulating the contraction of atrial muscles (100). Ang II stimulates auto-CaT by activating IP3R and CaMKII, inducing an abnormal release of SR Ca^2+^ and cytoplasmic Ca^2+^ overload. This subsequently triggers DAD by activating the NCX (101, 102). In addition, the Ang Ⅱ type 1 receptor plays a regulatory role in atrial myocytes. It enhances the I_Ks_ while concurrently decreasing the density of I_Na_ and reducing Cx40 expression. This modulation is facilitated through a phospholipase C-protein kinase C (PLC-PKC) cascade reaction. Such alterations contribute to the shortening of the APD and significantly influence AER (103, 104). Taken together, Ang Ⅱ can participate in the process of AER through the integrated regulation of sodium, potassium, and calcium channels.

#### 2.3.3 NACHT, LRR, and PYD domain-containing protein 3 (NLRP3 inflammasome)

NLRP3 inflammasome is a key inflammatory signaling pathway regulating innate immunity (105). The specific activation of the NLRP3 inflammasome can promote an abnormal release of SR Ca^2+^, leading to the production of DAD and resulting in ectopic myocardial discharges. It activates Ca^2+^-sensitive elements such as calcineurin (CaN) and CaMKⅡ by stimulating an increase in intracellular Ca^2+^ levels. Subsequently, the process activates and phosphorylates RYR2 and phospholamban, which enhances the release and reuptake of SR Ca^2+^. This, in turn, creates a positive feedback loop that further activates the NLRP3 inflammasome and sustains AER. Simultaneously, overactive NLRP3 signaling enhances I_Kur_ and shortens the AERP. These effects promote both the development and maintenance of AF (106–108).

#### 2.3.4 TNF-α

TNF-α, synthesized and secreted by macrophages and lymphocytes, is an endogenous mediator of inflammation. It participates in various cellular processes, including inflammatory and immunomodulatory responses, as well as growth inhibition (109). Significantly elevated levels of TNF-α in the plasma and left atrial tissue of patients with AF indicate its involvement in the pathogenesis of AF (110). TNF-α upregulates DNA methyltransferases, which enhances methylation of the SERCA2a promoter. This downregulates SERCA2a expression and impairs Ca^2+^ reuptake into the SR. As SERCA2a levels decrease, increased activity of the NCX occurs because of its competitive mechanism with SERCA2a, leading to the induction of DAD. In addition, TNF-α promotes AER by inducing an increase in the I_to_ and a decrease in I_Ca, L_, thereby shortening APD20 and APD50 (111, 112). In conclusion, the involvement of TNF-α in AER primarily manifests through the DAD phenomenon, which arises from increased SR Ca^2+^ leakage and reduced SERCA2a levels.

#### 2.3.5 Interleukin

IL is a lymphokine that interacts between immune cells, and it plays an important role in intercellular messaging, immune cell activation, and regulation. Several ILs, such as IL-2 (113), IL-6 (114), IL-18 (115), and IL-37 (116), showed elevated serum tests in patients with AF, suggesting their importance in the AF process.

IL-2, the first human interleukin to be identified, typed, and purified, is produced mainly by activated T lymphocytes. It is associated with recurrence after catheter ablation in patients with AF (117). IL-2 decreases CaT amplitude, while increasing diastolic Ca^2+^ levels and the CaT time constant. These changes increase the Na^+^-Ca^2+^ exchange current (I_NCX_), leading to DAD. This suggests that IL-2 alters myocardial electrical remodeling primarily through its effects on Ca^2+^ kinetics (118).

IL-6 is a pleiotropic cytokine with multiple biological activities that mediate proinflammatory responses and protect cells (119). During systemic inflammatory responses, elevated IL-6 levels inhibit the connexins Cx43 and Cx40. IL-6 also prolongs both the CaT alternans and the CaT refractory period. These alterations result in atrial Ca^2+^ handling abnormalities. Local imbalances in myocardial electrical activity lead to an increased susceptibility to cardiac alternans, which rapidly triggers AER (120). Moreover, IL-6 enhances miR-21 expression by inducing the phosphorylation of the signal transducer and activator of transcription 3 (STAT3), thereby activating cardiac fibroblasts (CF) and promoting atrial fibrosis (121). In addition, the upregulation of miR-21 expression contributes to a decrease in I_Ca, L_ density, indirectly participating in AER.

IL-10 is a potent anti-inflammatory cytokine with regulatory activity released by T cells and synthesized in several organs. It downregulates cell-mediated and cytotoxic inflammatory immune responses (122). Serum IL-10 levels are markedly reduced in patients with AF. Restoring IL-10 levels may ameliorate AF symptoms, likely by inhibiting proinflammatory cytokines such as TNF-α and IL-6α (123).

IL-18 is a proinflammatory cytokine produced mainly by macrophages and monocytes. IL-18 mediates the secretion of diverse cytokines and chemokines through the stimulation of activated T cells and proliferation of NK cells (124). A case–control study revealed that IL-18 levels were notably elevated in patients with AF compared with those in sinus rhythm. Furthermore, IL-18 concentrations were significantly higher in individuals with persistent AF than in those with paroxysmal AF. Consequently, IL-18 may be a more prominent inflammatory marker in AF than others (115). However, the exact mechanism by which IL-18 contributes to the development of AF remains unclear.

In conclusion, the involvement of ILs in AER is primarily linked to abnormal Ca^2+^ handling. While various ILs have been identified as closely associated with AF, research on their direct effects on myocardial electrical remodeling remains limited.

#### 2.3.6 C-reactive protein

C-reactive protein (CRP) is a non-specific marker for diagnosing inflammation and is an evolutionary conserved protein playing a role in innate immune signaling (125). Studies have shown that CRP levels are elevated in patients with non-operative AF, and higher CRP concentrations are linked to an increased rate of AF recurrence after resuscitation (126, 127). P-wave dispersion (Pd) is a non-invasive electrocardiographic marker that may indicate prolonged intra-atrial and interventricular conduction times and discontinuous, inhomogeneous propagation of sinus impulses. The interaction between high-sensitivity C-reactive protein (hs-CRP) and AF may be mediated by Pd, leading to intra-atrial conduction heterogeneity and dispersion of the atrial refractory period, thereby creating conditions conducive to AF (128). A genetic association study between CRP gene polymorphisms and AF revealed that CRP significantly upregulated I_Ca, L_ levels in atrial myocytes. But it did not affect other ion flows or precollagen-encoding genes in atrial fibroblasts. Thus, it is speculated that the mechanism linking increased CRP levels to the onset of AF might be related to Ca^2+^ overload (129).

#### 2.3.7 Matrix metalloproteinase

Matrix metallo proteinase (MMPs), belonging to the metalloproteinase family, are integral in several physiological processes, including angiogenesis and cell growth. Their expression levels could potentially predict recurrence following AF resuscitation, thus establishing MMPs as promising biomarkers for AF diagnosis and management (130). MMPs exert an indirect effect on AER by influencing inflammatory responses, oxidative stress, and myocardial fibrosis (131, 132). Notably, the levels of MMP-1, -3, -7, and -9 are significantly elevated in the atrial tissues of patients with AF. Inhibiting MMP activity has been shown to substantially reduce AF susceptibility, suggesting that heightened MMP activity in the atrium is a contributing factor to AF (133, 134). In a Beagle dog model of burst stimulation-induced AF, MMPs contribute to the process of atrial fibrosis. They promote fibroblast proliferation and collagen formation by upregulating B-cell lymphoma-2 (BCL-2) and downregulating BCL-2-associated X protein expression levels (135).

### 2.4 Oxidative stress and AER

**Figure 4:**
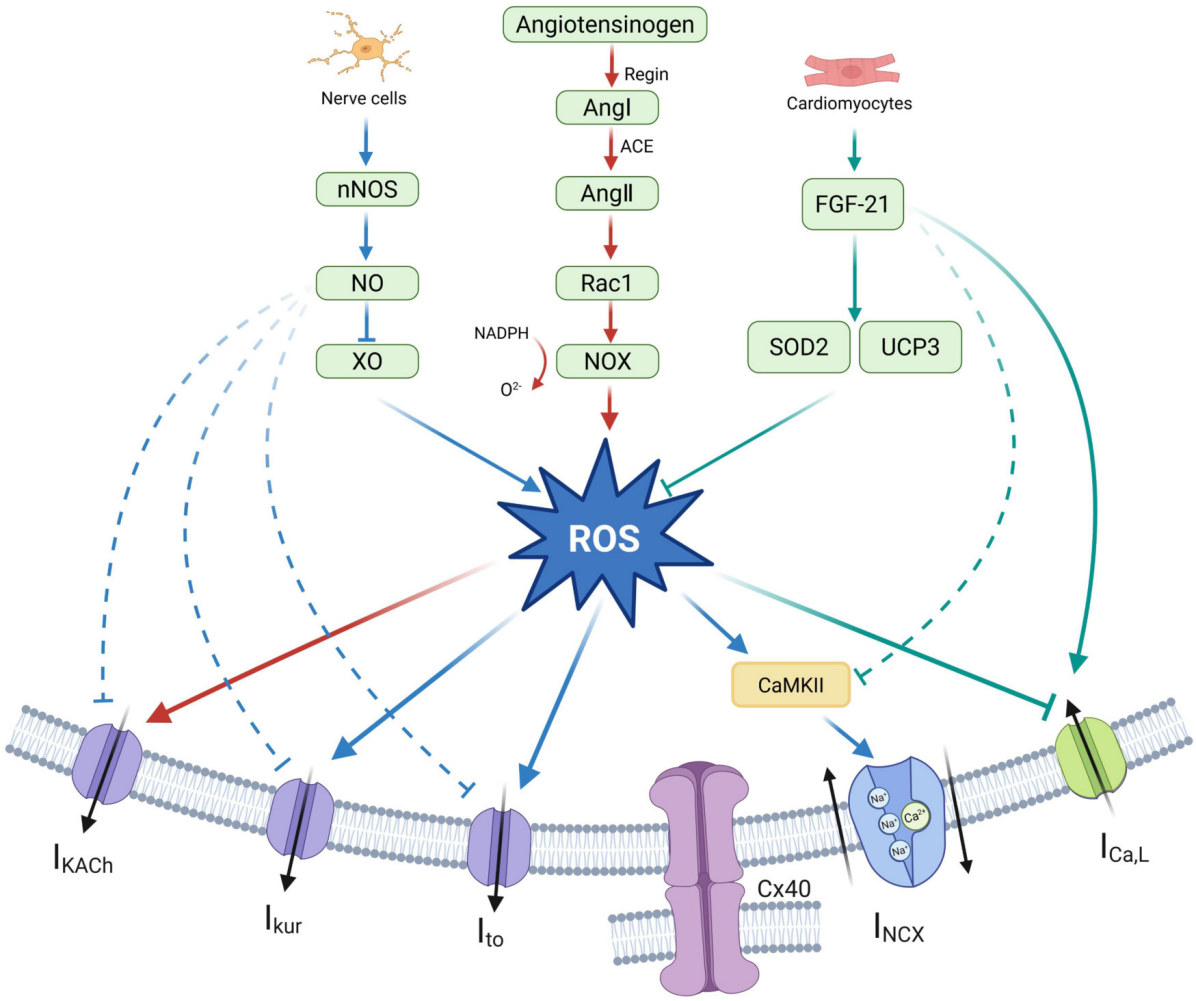
Oxidative stress and atrial electrical remodeling. Oxidative stress significantly impacts atrial electrical remodeling, primarily because of the overproduction of ROS. Critical in regulating this ROS generation are enzymes and factors such as XO, NOS, NOX, and FGF-21. By Figdraw (http://www.figdraw.com).

Oxidative stress is characterized by a disruption in the balance of intracellular redox reactions, leading to an excessive production of free radicals (136). This imbalance can cause significant damage to cellular structures and functions. It is primarily associated with the overproduction of ROS, which can inflict cellular damage and lead to cell death. This damage includes harm to DNA, proteins, cell membranes, and organelle functions, all of which are closely linked to the development of cardiovascular diseases (137). A key source of ROS is mitochondrial dysfunction. In addition, nicotinamide adenine dinucleotide phosphate oxidase (NOX), xanthine oxidase (XO), and nitric oxide synthase (NOS) are significant contributors to ROS production in atrial tissue. These factors play a crucial role in AER, predominantly by impacting Ca^2+^ homeostasis and energy metabolism (Figure 4).

#### 2.4.1 NOX and AER

NOX is a class of proteins that transfer electrons across biological membranes, catalyzing the transfer of electrons from nicotinamide adenine dinucleotide phosphate (NADPH) to O_2_ molecules. It is a major source of ROS in the body and plays a key role in driving oxidative stress within the cardiovascular system. A prospective study indicated that NOX is a key mediator in atrial oxidative stress, leading to postoperative AF (138). It is an independent predictive factor for postoperative AF. As a significant source of ROS, the involvement of NOX in AER is primarily associated with disturbances in ion balance caused by Ang Ⅱ mediation and abnormalities in energy metabolism. NOX1 elevates Ang Ⅱ by mediating Ca^2+^ signaling, which, in turn, increases NOX activity, stimulating the production of ROS in cardiac myocytes. Hydrogen sulfide (H_2_S) can downregulate Ang Ⅱ-induced atrial Kv1.5 expression by inhibiting the NOX4-ROS signaling pathway (139, 140). In addition, NOX-derived ROS can activate protein kinase C (PKC) via oxidative mechanisms. This upregulates ROS-dependent I_KACh_, thereby promoting AER (141).

#### 2.4.2 XO and AER

XO is a pivotal enzyme in purine catabolism, playing a key role in the production of uric acid. It contributes to oxidative stress through mechanisms such as calcium signaling. Cells treated with XO activate the NLRP3 inflammasome and its associated downstream proinflammatory signals, including ILs like IL-1β and IL-18 (142). Following cellular injury, the availability of xanthine and hypoxanthine increases. XO then uses oxygen as a potential electron acceptor to generate ROS, leading to the induction of oxidative stress (143). ROS exacerbates SR Ca^2+^ leakage and DAD by promoting the formation of oxidized CaMKII (ox-CaMKII) and enhancing RyR2 phosphorylation. In addition, XO downregulates Cx40 expression, disrupting its distribution in the atrium. XO may also affect atrial electrical remodeling indirectly by influencing NOX activity. In turn, it induces the development of AF (144, 145).

Mitochondrial transcription factor A (TFAM) and nuclear respiratory factor-1 (NRF-1) are key activators of mitochondrial transcription. Mitofusin1 (Mfn1) is a mitochondrial membrane protein that acts as a mediator of mitochondrial fusion (146). Allopurinol, an inhibitor of XO, significantly prolongs interatrial conduction time and atrial-effective refractory period dispersion (AERPD). Allopurinol improves mitochondrial function by downregulating TFAM, NRF-1, and Mfn1. It also reduces AF susceptibility by lowering Ca^2+^ overload, achieved through reducing I_Ca, L_ density and CaT amplitude (147).

#### 2.4.3 NOS and AER

Endocardial NOS expression and nitric oxide (NO) bioavailability are significantly reduced in patients with AF (148). Endogenous NO is synthesized from L-arginine by different isoforms of NOS. NO facilitates the maintenance of mitochondrial homeostasis and exerts an anti-inflammatory effect through specific inhibition of NLRP3 inflammatory (149). The AF-induced reduction in NO levels increases outward potassium currents through Kv1.5 and Kv4.3 channels. This shortens the APD and AERP. In addition, it triggers Na^+^-dependent Ca^2+^ overload, which contributes to AER (150, 151). Neuronal nitric oxide synthase (nNOS) is a crucial component of the antioxidant system. Its deficiency leads to a significant increase in superoxides mediated by XO, which, in turn, inhibits the activity of xanthine oxidoreductase. In addition, nNOS regulates the SR Ca^2+^ cycle, thereby inhibiting the excitation–contraction coupling in cardiac muscles (152).

#### 2.4.4 Fibroblast growth factor 21 and AER

Fibroblast growth factor 21 (FGF-21) is a novel regulator of oxidative stress in humans. It plays an important role in cardiovascular disease through endocytosis. FGF-21 upregulates LTCC expression and downregulates ox-CaMKⅡ and p-RyR2 expression to inhibit the aberrant release of SR Ca^2+^, thereby reversing Ang Ⅱ-induced APD shortening. It also directly induces the expression of antioxidant genes such as superoxide dismutase 2 and uncoupling protein-3 (UCP3). This reduces ROS levels, alleviates oxidative stress, and decreases AF susceptibility (153). Clinical evidence showed that FGF-21 levels were significantly elevated in the atrial tissue of patients with AF, and its expression level was positively correlated with the degree of atrial fibrosis (154). Based on these observations, the elevated FGF-21 levels in AF are hypothesized to be a compensatory response.

### 2.5 Autonomic neural remodeling and AER

The nervous system stands as one of the most intricate systems within an organism. It orchestrates the essential functions of sensing, processing, and responding to a vast array of information on both the internal and external environments of the organism. Within this complex system, the ANS emerges as a pivotal component of the neurohumoral network, playing a crucial role in regulating the heart's electromechanical activities. The ANS exerts its influence on cardiac electrophysiology primarily through the release of neurotransmitters such as acetylcholine (Ach), dopamine (DA), and norepinephrine (NE). The structure of the ANS is predominantly composed of two fundamental divisions: the sympathetic nervous system (SNS) and the parasympathetic nervous system (PNS). Both these systems harmoniously interact to maintain physiological equilibrium. Among the critical nerves of the SNS is the sympathetic nerve (SN), and for the PNS, the vagus nerve (VN) is particularly essential. Each plays a significant role in the nuanced modulation of the body's involuntary functions, particularly in the context of cardiac regulation.

The balance between sympathetic and vagal nerve activities is crucial for maintaining ionic homeostasis in cardiac myocytes. Simultaneous discharge of the SN and VN is the most common trigger for paroxysmal AF (155). AF occurs with an uneven distribution of atrial autonomic nerves and autonomic remodeling. Autonomic remodeling activates the SN and VN and disrupts the sympathovagal balance. CaT is essential for cardiac muscle contraction. SN activation promotes CaT, increasing cardiac automaticity and spontaneous activity. Conversely, VN activation upregulates Na^+^-K^+^-ATPase. This shortens the AERP and APD, can trigger EAD, and facilitates the maintenance or recurrence of AF (156–159).

The ANS plays a pivotal role in the initiation and perpetuation of AF through a synergistic interplay of triggered, electrical, and structural loops (160). In the context of prolonged rapid atrial pacing, the activation of the SN leads to the release of NE from its nerve endings. NE, in turn, stimulates calcium channels in the myocardial membrane, causing an overload of intracellular Ca^2+^ and aberrant Ca^2+^ release from the SR. This cascade increases ectopic pacing sites within atria, thereby elevating the risk for AF; these are known as the triggered loops. During episodes of rapid atrial rate, activation of VN and subsequent release of Ach lead to the activation of atrial I_KACh_. This action consequently downregulates I_Ca, L_, leading to a reduction in both the APD and AERP. This sequence helps sustain the self-perpetuating circuitry of AF, forming the electrical loop. Structural loops are intricate processes wherein various cardiovascular conditions, including AF, heart failure, and heart valve disease, induce structural remodeling of the myocardium. This remodeling further aggravates AF. Prolonged episodes of AF are typically associated with myocardial fibrosis and an increased dissociation of electrical conduction between the epicardial layer and the endocardial bundle. These changes foster the development of foldback circuits, contributing to the persistence of AF. Collectively, these three types of loops—triggered, electrical, and structural—establish a positive feedback mechanism that significantly contributes to both the development and maintenance of AF (161, 162).

The molecular mechanisms underpinning cardiac ANS transmission are primarily governed by adrenergic and cholinergic pathways. The ANS influences AER through the release of neurotransmitters such as Ach, epinephrine (EPI), and isoprenaline (ISO). Ach, a cholinergic neurotransmitter predominantly released by the VN, plays a crucial role in various physiological functions, including glandular secretion and heart rate modulation. Ach directly activates I_KACh_ while inhibiting I_Kr_ and I_Ca,L_. These effects shorten the APD and AERP. Ach also induces aberrant SR Ca^2+^ release, triggering Ca^2+^ overload. On the other hand, Ach also reduces the conduction rate of electrical signals between atrial cells by inhibiting intercellular gap junction communication and maintains intra-atrial refractoriness (163, 164). EPI, primarily released by the SN and secreted by the adrenal glands, is integral to cardiac excitation and blood glucose elevation. Its role in cardiac electrical remodeling is closely associated with Ca^2+^ overload and potassium channel abnormalities. EPI promotes Ca^2+^ binding to calmodulin, thereby activating CaMKII. This enhances Ca^2+^ influx and promotes spontaneous Ca^2+^ release from the SR. This can lead to EAD and DAD (165). Moreover, EPI shortens the APD by enhancing I_KACh_ and I_Ks_ (166, 167). Another study found that the alpha1 adrenergic receptor (α1AR) inhibited IK1 by activating the Src tyrosine kinase and PKC pathways. Localized IK1 inhibition may trigger arrhythmias induced by ectopic depolarization, whereas a more generalized reduction in IK1 may inhibit AF by prolonging APD (168). In chronic AF cells, ISO modifies the APD by increasing I_Ks_ density, which shortens APD90, and decreasing I_to_ density, which prolongs APD20. These effects are associated with the activation of beta1 adrenergic receptors (β1AR), a process mediated by CAF (169, 170).

**Figure 5:**
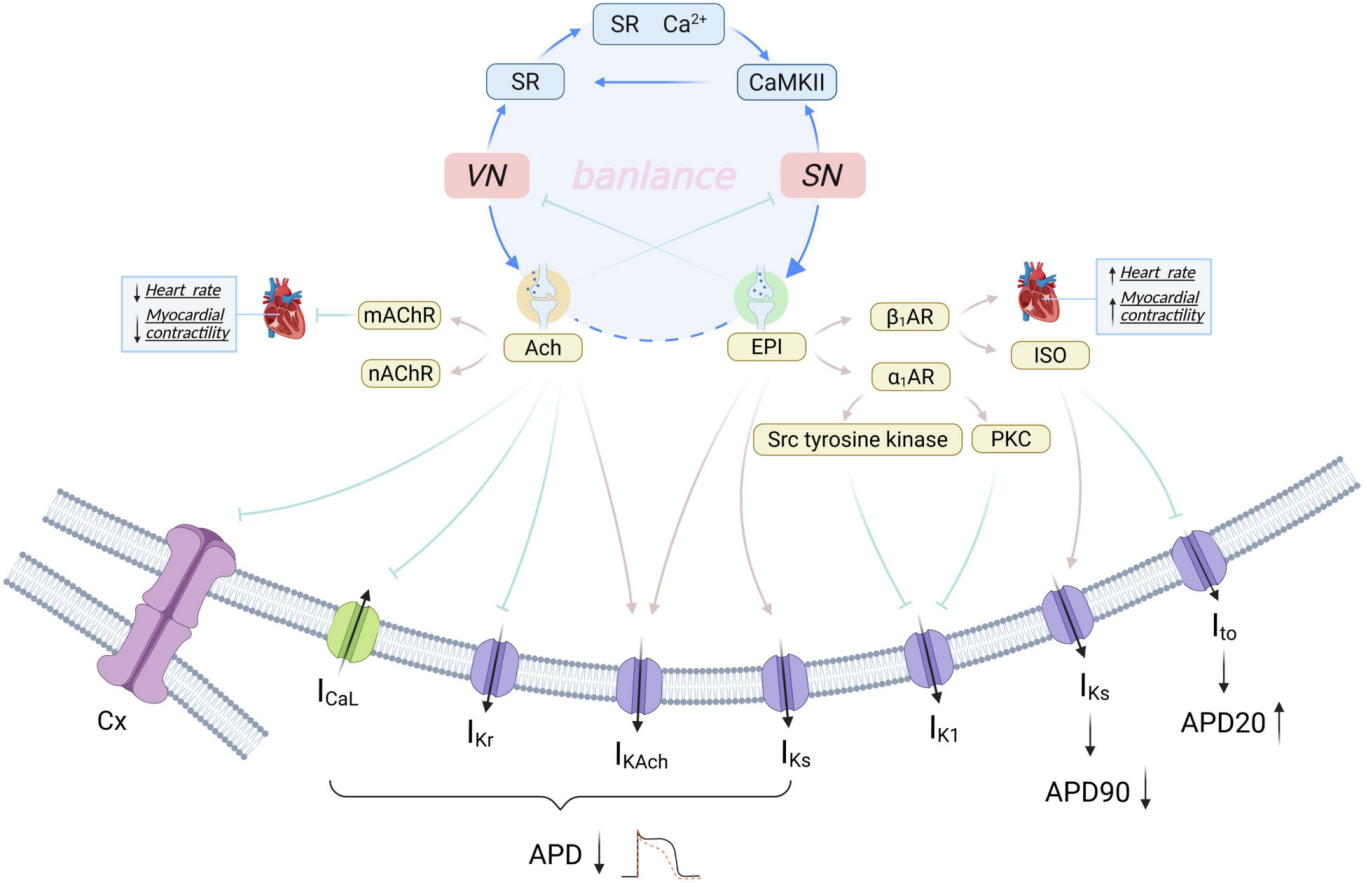
Autonomic nervous system remodeling and atrial electrical remodeling. The ANS is a crucial structure in the nervous system for regulating the electromechanical activities of the myocardium. It functions comprehensively by regulating the release of neurotransmitters such as Ach and EPI through the SN and VN, playing a vital role in regulating the ionic homeostasis of myocardial cells. By Figdraw (http://www.figdraw.com).

In summary, the development and persistence of AF are intricately associated with a synergistic interaction between autonomic and electrical remodeling. A characteristic feature of autonomic nerve remodeling in this context is the hyperactivation of both the SN and VN, coupled with a dysregulation in the sympathovagal balance. This remodeling of the autonomic nerves, in conjunction with electrical remodeling, forms a self-perpetuating cycle that acts as a crucial factor in the maintenance and exacerbation of AF. This complex interplay underscores the importance of addressing both autonomic and electrical aspects in the management and treatment of AF (Figure 5) (171).

### 2.6 Myocardial fibrosis and AER

**Figure 6:**
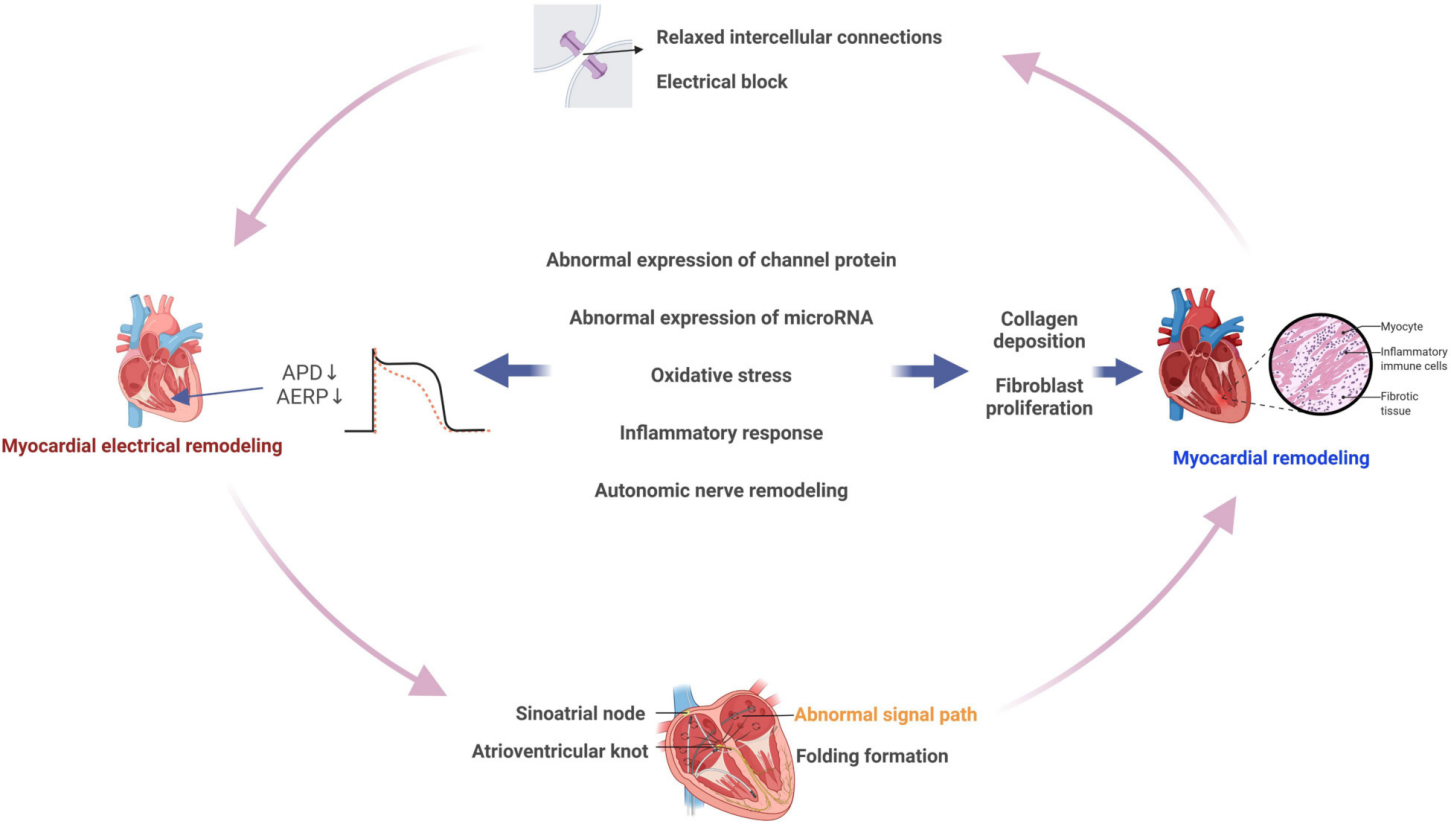
Myocardial fibrosis and atrial electrical remodeling. Myocardial electrical remodeling and myocardial structural remodeling influence and promote each other. Myocardial structural remodeling leads to the relaxation of intercellular connection and electrical block, promotes the formation of reentry, strengthens the damage of myocardial cells, and further aggravates myocardial structural remodeling. Ion channel change, miRNA expression, oxidative stress, inflammatory response, and autonomic nervous system remodeling are the common factors influencing myocardial electrical remodeling and myocardial structural remodeling. By Figdraw (http://www.figdraw.com).

Myocardial fibrosis is characterized by abnormal proliferation of fibroblasts and excessive collagen deposition in the extracellular interstitium of cardiomyocytes (172). Long-term AF exacerbates atrial fibrosis because of its persistent uneven electrical conduction, and the formation of atrial fibrosis promotes the maintenance of AF. Therefore, myocardial fibrosis (a key aspect of structural remodeling) and electrophysiological/ion channel alterations (hallmarks of electrical remodeling) are intricately interconnected and mutually influential (173). A growing number of studies have shown that the underlying mechanisms of myocardial fibrosis are closely related to altered ion channels, abnormal miRNA expression, inflammatory response, and oxidative stress (Figure 6) (174, 175).

#### 2.6.1 Ion channel alterations and myocardial fibrosis

Calcium channel dysregulation is the primary mechanism by which ion channel alterations induce myocardial fibrosis to promote AF. In a profibrotic environment, upregulation of I_K1_ in atrial fibroblasts hyperpolarizes the resting membrane potential. This increases the driving force for Ca^2+^ entry. Increased Ca^2+^ triggers downstream signaling cascades such as mitogen-activated protein kinases (MAPKs) and NF-κB, which elicits responses such as fibroblast activation and cardiomyocyte hypertrophy to promote myocardial fibrosis (176, 177). The transient receptor potential canonical-3 (TRPC3) channel is Ca^2+^-permeable. NFAT upregulates TRPC3 expression by downregulating miRNA-26. Increased TRPC3 promotes Ca^2+^-dependent phosphorylation of extracellular signal-regulated kinase, thereby inducing fibroblast proliferation and differentiation (178).

#### 2.6.2 MiRNAs and myocardial fibrosis

MiRNAs can act as activators or inhibitors of profibrotic factors. Maintaining a balance between the antifibrotic and profibrotic effects of miRNAs is essential for controlling myocardial fibrosis. Dicer, being a key RNAseⅢ endonuclease responsible for miRNA production and maturation, plays a significant role. Its deficiency leads to rapid, pronounced ventricular dilation, cardiomyocyte hypertrophy, and myofibrillar disorganization. This underscores the critical link between miRNA processing and myocardial fibrosis (179). In recent years, an increasing number of studies have focused on understanding how miRNAs mitigate AF by modulating myocardial fibrosis. MiRNA-29β is a critical regulator of atrial fibrosis. Its upregulation in rats diminishes atrial fibrosis and AF by suppressing TGF-β1 expression (180). MiR-124-3p is markedly overexpressed in patients with AF and can modulate the Wnt signaling pathway via its target gene AXIN, thereby enhancing the proliferation of CF (181). Downregulation of miR-133 and miR-590 triggers a signaling cascade mediated by TGF-β and TGF-βRII. This increases collagen production and deposition in the myocardium, leading to fibrosis. Ultimately, this contributes to atrial remodeling and fibrillation (182, 183). By directly targeting TGF-β in fibroblasts, miR-324-3p inhibits fibroblast proliferation via the phosphoinositide 3-kinase (PI3 K)/AKT pathway, thereby playing a therapeutic role in AF (184).

#### 2.6.3 Inflammatory response and myocardial fibrosis

The RAS plays a key role in cardiac structural remodeling. Activation of the angiotensin-converting enzyme–angiotensin Ⅱ–angiotensin Ⅱ type 1 receptor (ACE–Ang Ⅱ–AT1) axis leads to an increase in cardiac load, thereby triggering myocardial structural remodeling. The ACE inhibitor enalapril alleviates the persistence of AF by delaying conduction through the inhibition of interstitial fibrosis and Cx43 overexpression (185). Ang Ⅱ triggers the phosphorylation of tyrosine in STAT3 through an RAS-related C3 botulinum toxin substrate 1 (Rac1)-dependent mechanism and enhances the expression of activin-A along with its specific downstream element, activin receptor-like kinase 4. This process leads to an increase in protein synthesis within atrial myocytes and fibroblasts, thereby playing a pivotal role in the structural remodeling of the atria (186, 187). The ACE2–Ang-(1-7)–Mas axis acts as an inhibitor of Ang Ⅱ. It mitigates Ang Ⅱ-induced collagen synthesis and myocardial fibrosis by inhibiting the intermediate-conductance Ca^2+^-activated K^+^ (K_Ca3.1_) channels. This shows that inhibition of the RAS has a potential therapeutic role in AF (188, 189).

TGF-β is one of the most potent profibrotic growth factors. It can regulate collagen production and deposition and induce the expression of genes associated with the development of myocardial fibrosis, such as connective tissue growth factor (CTGF). CD44 is a transmembrane receptor for hyaluronan and plays a crucial role in coordinating various cellular processes, including proliferation, migration, and differentiation. This receptor is significantly involved in the modulation of myocardial fibrosis, highlighting its importance in cellular regulation and tissue health (190). In models overexpressing TGF-β, large amounts of p-STAT3, which binds to the type I collagen promoter, are produced and profibrotic CD44 production is increased. This exacerbates myocardial fibrosis and AF susceptibility. In contrast, it was found that when atrial fibroblasts were treated with an anti-CD44 blocking antibody, TGF-β1-induced collagen transcriptional activity was attenuated and AF development was inhibited (191, 192). Furthermore, platelet-derived TGF-β1 promotes Ang Ⅱ-induced atrial fibrosis and AF (193).

PDGF-D and TGF-β1 reciprocally amplify each other's effects, resulting in a synergistic impact (194). Specifically, PDGF-D enhances the expression of MMP-1, MMP-2, and MMP-9 in fibroblasts by stimulating the synthesis of type I collagen. This action contributes to the increased proliferation and migration of fibroblasts. Upregulation of PDGF-AA in atria also induces atrial fibrosis and enhances AF induction in normal hearts, whereas neutralizing PDGFR-α-specific antibodies attenuate atrial fibrosis in pressure-overloaded hearts (194).

#### 2.6.4 Oxidative stress and myocardial fibrosis

Oxidative stress is a key regulator in the process of myocardial fibrosis. The elevation of oxidative stress levels, driven by the release of certain inflammatory mediators, fosters the production of ROS. These excessive ROS act as potent proinflammatory signals. They not only mediate the effects of extracellular signals such as TGF-β and Ang II but also activate numerous intracellular proteins, enzymes, and transcription factors. This activation leads to an increase in collagen synthesis and cellular hypertrophy within the myocardium. Furthermore, oxidative stress modulates the activity of MMP-2 and MMP-9. These enzymes are crucial for extracellular matrix turnover, and their dysregulation contributes to the progression of myocardial fibrosis (195). Bromodomain-containing protein 4 (BRD4), a member of the bromodomain and extraterminal family, is observed to have increased levels in hypertrophied myocardial cells. Blocking BRD4 can lead to a reduction in the production of ROS, which, in turn, significantly lowers the expression of profibrotic genes such as TGF-β1, collagen I, and collagen Ⅲ (196). Thus, targeting the reduction of ROS generation as a strategy to alleviate myocardial fibrosis is a critical therapeutic approach in the treatment of AF.

#### 2.6.5 Autonomic remodeling and myocardial fibrosis

Presently, research into the impact of autonomic nerve remodeling on the onset and progression of myocardial fibrosis remains somewhat scarce. Excessive and rapid activation of β-adrenergic receptors (β-AR) in the heart can activate IL-18 inflammasomes, which, in turn, initiate a cytokine cascade and macrophage infiltration. This process contributes to the development of pathological myocardial fibrosis (197). Moreover, administering high doses of isoproterenol in rats leads to ischemic infarction in atria, which increases the variability of atrial fibrosis and its conduction properties. This effect consequently heightens the susceptibility of rats to AF (198).

In conclusion, myocardial structural remodeling and electrical remodeling are interdependent, each influencing the other. Myocardial fibrosis disrupts electrical conduction continuity through collagen deposition and scar formation. In addition, by altering the expression and distribution of connexins like Cx40, Cx43, it increases electrical conduction inhomogeneity and impedes impulse propagation. This process contributes to myocardial electrical remodeling by inducing spontaneous phase 4 depolarization, which shortens the APD (199, 200). Conversely, myocardial electrical remodeling exacerbates the development of myocardial fibrosis by affecting cytokine secretion and signal transduction pathways, creating a vicious cycle. Myocardial fibrosis arises when the production of collagen surpasses its degradation, a condition resulting from an intricate interplay of various factors. This includes alterations in cardiomyocyte ion channels, miRNA regulation, inflammatory responses, oxidative stress, and autonomic nerve remodeling. These factors interact to form a complex network that directly and indirectly promotes both AER and structural remodeling, thus playing a crucial role in the development of AF.

## 3 Natural drugs and active ingredients targeting the inhibition of AER for treating AF

### 3.1 Alkaloids

Alkaloids are a class of naturally occurring nitrogen-containing organic compounds that typically exhibit basic properties, with nitrogen atoms often incorporated into heterocyclic structures. They serve as key components responsible for the pharmacological activity of many medicinal plants. Based on their biosynthetic precursors and core chemical structures, alkaloids can be classified into organic amines, quinolizines, isoquinolines, and others. Alkaloids often exhibit significant physiological or toxic effects such as analgesia, anti-inflammation, and antiarrhythmia (201).

#### 3.1.1 Organic amines

##### 3.1.1.1 Colchicine

**Figure 7:**
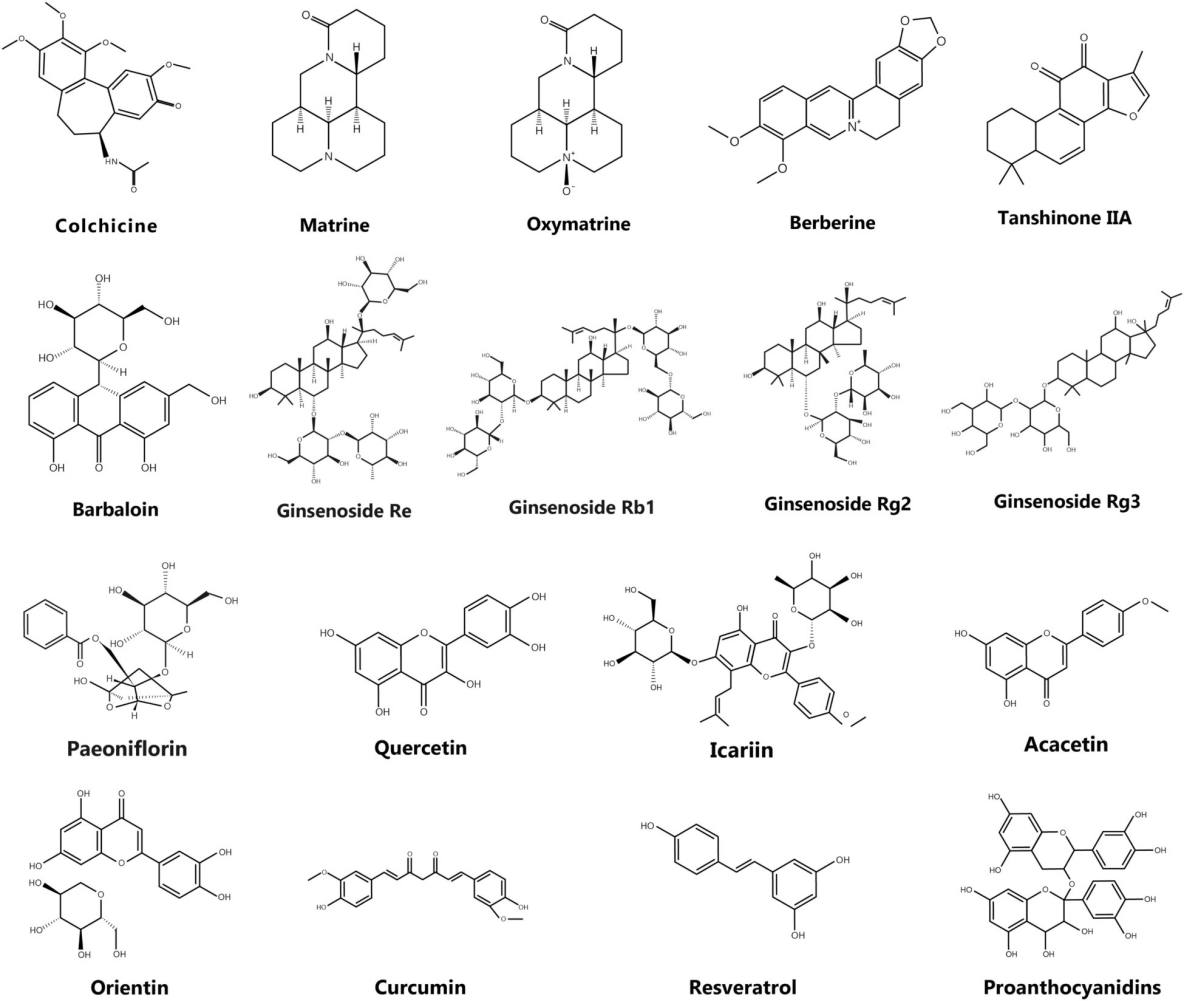
The chemical structure of active components in natural medicines.

Colchicine (C_₂₂_H_₂₅_NO_₆_), a natural alkaloid originally extracted from plants of the *Colchicum* genus in the lily family (Figure 7), is widely used for its anti-inflammatory properties. Clinically, it has been employed to prevent AF following cardiac surgery and pulmonary vein isolation procedures (202).

Colchicine may exert its anti-arrhythmic effects through the modulation of Ca^2+^ handling, inflammation, and myocardial fibrosis. It inhibits the phosphorylation of calcium channels, thereby reducing Ca^2+^ overload and the associated risk of tachyarrhythmias (203). In terms of anti-inflammatory action, colchicine prevents AF by significantly attenuating the phosphorylation of signaling molecules such as p38 MAPK, AKT, JNK, and NF-κB. In a rat model of aseptic pericarditis, this effect manifested as reduced neutrophil infiltration and decreased expression of proinflammatory cytokines such as IL-6, TGF-β, and TNF-α (204). With regard to its antifibrotic effects, colchicine inhibits myocardial fibroblast activation by suppressing the TGF-β1 signaling pathway and downregulating type I and type III collagen synthesis in rats (205). In addition, studies using a transverse aortic constriction (TAC) model in mice confirmed that colchicine activates the Sirtuin 3 (SIRT3) protein, thereby reducing oxidative stress and myocardial fibrosis, and consequently alleviating stress-induced cardiac remodeling (206). From the perspective of structure–activity relationship (SAR), the tricyclic skeleton, the C-7 methoxy group, and the C-10 amide group of colchicine constitute its key pharmacophores for binding to tubulin and inhibiting inflammasome activation. This mechanism potentially underlies its antifibrotic effects (207, 208).

#### 3.1.2 Quinolizidine alkaloids

##### 3.1.2.1 Matrine

Matrine, with the molecular formula of C_15_H_24_N_2_O (Figure 7), is an alkaloid made from the dried roots, plants, and fruits of the leguminous plant *Sophora flavescens* Ait. It is typically isolated using ethanol and other organic solvents and serves as the primary pharmacologically active compound in *Sophora flavescens*. *In vivo* studies have demonstrated that Matrine effectively counters AF by modulating the M3 receptor (M3-R) and LTCC. Matrine upregulates the α1C/Cav1.2 subunit protein (without affecting its mRNA) and increases I_Ca, L_ density. Concurrently, it downregulates M3-R and the M3 receptor-mediated K^+^ current (IKM3). These combined effects markedly extend the APD and AERP (209). From a SAR perspective, the structural motif “O = C = N–C–C–C–N” shared by matrine and its analogues is considered the key pharmacophore responsible for the positive inotropic effects of these alkaloids and their potential activation of calcium channels (210). In a TAC surgery pressure overload mouse model, matrine can downregulate the levels of multiple inflammatory factors and the expression of Cx40 and Cx43 by inhibiting the Wnt3a/β-catenin signaling pathway. It inhibits TAC-induced atrial electrical and structural remodeling, thereby reducing susceptibility to AF (211). Furthermore, matrine inhibits the transformation of rat myocardial fibroblasts into myofibroblasts by reducing TGF-β1 and MMP-9 expression through decreased levels of type I and type III collagen, thereby enhancing myocardial uniformity and atrial conduction velocity (212). Consequently, matrine combats AF by regulating M3-R, LTCC, and antifibrotic processes, collectively impeding electrical remodeling.

##### 3.1.2.2 Oxymatrine

Oxymatrine has the molecular structure C_15_H_24_N_2_O_2_ (Figure 7). It is an alkaloid isolated from the Sophora species of the Fabaceae family, known for its tissue-protective, anti-inflammatory, and antiviral therapeutic actions (213). Its mechanism of action in organ tissue protection primarily relates to its anti-inflammatory, antioxidative stress, antifibrotic, and metabolic regulatory functions, which collectively lead to optimal therapeutic effects (214). Electrophysiological research indicates that oxymatrine inhibits sodium and Ca^2+^ currents in isolated rat ventricular myocytes in a concentration-dependent manner (215). Oxymatrine is a promising antiarrhythmic agent. It normalizes abnormally shortened or prolonged APD in arrhythmic rats by modulating I_Ca, L_ and I_K1_ and enhancing I_to_ (216).

Oxymatrine is chemically similar to matrine and both compounds have the potential for antiarrhythmic effects, primarily achieved by inhibiting sodium and calcium channels.

#### 3.1.3 Isoquinoline alkaloids

##### 3.1.3.1 Berberine

Berberine is an alkaloid isolated from the traditional Chinese medicine Huanglian. It is a non-basic quaternary ammonium salt of the phenylisoquinoline alkaloids with anti-arrhythmic and anti-inflammatory properties, which has broad application prospects in the prevention and treatment of cardiovascular diseases (Figure 7) (217, 218). Clinical studies have shown that berberine can effectively reduce the occurrence of postoperative AF after coronary artery bypass grafting. There is no significant difference between its effect and that of amiodarone on the conversion of AF to sinus rate and the maintenance of normal sinus rhythm (219). Berberine inhibits myocardial fibrosis by activating the AMP-activated protein kinase (AMPK)-PPARα signaling pathway, thereby improving AF in a rabbit model (220). By inhibiting potassium channels and stimulating Na^+^-Ca^2+^ exchange, berberine increases coronary blood flow. This helps prevent AF following acute myocardial infarction. In addition, berberine prolongs the APD and AERP in rabbit atrial myocytes by blocking multiple ion channels, including K^+^ channels, thereby inhibiting Ach-induced AF (221). From an SAR perspective, the core pharmacophore of berberine includes an isoquinoline skeleton, C-2/C-3 methoxy groups, and a C-7 quaternary ammonium cation. These elements collectively form a rigid planar aromatic system. This enables simultaneous interaction with multiple targets, including ion channels, inflammatory signaling proteins, and metabolic regulatory enzymes, forming the basis for its multifaceted cardioprotective effects (222).

### 3.2 Saponins

Saponins are a class of structurally complex glycoside compounds formed by the linkage of hydrophobic triterpenoid or steroidal aglycones to hydrophilic oligosaccharide chains via glycosidic bonds. Their amphiphilic structure enables them to readily form foam in water. Widely distributed throughout the plant kingdom, saponins exhibit diverse biological activities, including anti-inflammatory effects, immunomodulation, cholesterol-lowering properties, and cardiovascular protection (223).

#### 3.2.1 Ginsenosides

Ginsenosides, among the most potent active components extracted from ginseng, encompass types such as Rb1, Rb2, Rc, Rd, Re, and Rf (Figure 7). Their application in a wide range of diseases is attributed to their multitarget, multipathway effects and low incidence of side effects (224).

As the predominant ginsenoside, Rb1 indirectly improves AER through its anti-inflammatory and antimyocardial fibrosis effects. Electrophysiological studies show that Rb1 concentration-dependently inhibits I_Na_ and I_Na_ channels, leading to APD shortening. Furthermore, it alleviates high Ca^2+^-induced DAD in rabbit ventricular myocytes by downregulating RyR2 and upregulating SERCA protein levels (225). In a rat model of cardiac injury induced by aconitine, Rb1 significantly lowered levels of caspase-1, NLRP3, IL-1β, IL-18, and other cellular juxtaposition-associated factors. This indicates its cardioprotective role by inhibiting apoptosis and pyroptosis through anti-inflammatory pathways (226). From an oxidative stress perspective, Rb1 curtails mitochondrial ROS production and NADH dehydrogenase activity, thereby mitigating cardiac ischemia/reperfusion (I/R) injury in mice (227). Furthermore, by inhibiting the TGF-β1/Smad pathway, Rb1 alleviates myocardial fibrosis in rats. This is associated with a downregulated expression of type I collagen, Ang II, ACE, and AT1 receptors, demonstrating its therapeutic potential for AF (228).

Other research has delved into the mechanisms of different types of ginsenosides, such as Re, Rg2, Rg3, and Rd, in treating AF. Electromechanical alternans (EMA) is a proarrhythmic phenomenon characterized by the periodic beat-to-beat alternation of the APD and contraction amplitude at a constant stimulus frequency, being a significant factor in the pathogenesis of AF (229). Ginsenoside Re can inhibit EMA in atrial myocytes by enhancing the frequency of RyR channel openings, which promotes Ca^2+^ release from the SR (230). In a rat model of CaCl_2_-induced arrhythmia, Rg2 exerts antiarrhythmic effects by inhibiting Ca^2+^ inward flow through the suppression of CaMKⅡ*δ* phosphorylation (231). Rg3 demonstrates anti-inflammatory potential for treating NLRP3-related diseases by inhibiting lipopolysaccharide-induced NLRP3 inflammasome activation and IL-1β secretion in human and mouse macrophages (232). Rd inhibits α1AR activation and reduces Ca^2+^ influx in rat vascular smooth muscle cells via the receptor-operated calcium channel (ROCC) and the store-operated calcium channel (SOCC) (233).

### 3.3 Flavonoid compounds

Flavonoids are a class of phenolic secondary metabolites based on the 2-phenylchromenone (C6–C3–C6) skeleton (234) (Figure 7). This skeleton consists of two benzene rings (A-ring and B-ring) connected via an oxygen-containing heterocyclic ring (C-ring). Based on the oxidation state and saturation of the C ring, along with the linkage position of the B ring, they are primarily classified into subclasses such as flavonoids, flavanols, flavonones, isoflavones, flavanones, and anthocyanins. Flavonoids are renowned for their potent antioxidant, anti-inflammatory, and cardiovascular protective activities (235).

A SAR analysis revealed that the quantity and position of hydroxyl groups, 3′,4′-catechol groups, C2 = C3, and C4 = O groups in flavonoids may play a significant role in their cardioprotective activity (236).

#### 3.3.1 Quercetin

Quercetin and its derivatives are potentially valuable in the prevention of cardiovascular disease because of their cardioprotective effects (237).

Quercetin mainly exerts its effects on AER by inhibiting oxidative stress and myocardial fibrosis. In terms of inhibiting oxidative stress, quercetin reduces the activity of NADPH oxidase and the content of ROS, while increasing the content of endothelial-type nitric oxide synthase (eNOS) and the bioavailability of NO in rat (238). In improving myocardial fibrosis, quercetin protects mitochondrial function by modulating the sirtuin3 (SIRT3)/poly-ADP-ribose polymerase-1 (PARP-1) signaling pathway in hypertensive rats, thereby inhibiting oxidative stress and preventing myocardial fibrosis (239, 240). The compound is also effective in suppressing the expression of Ang Ⅱ-induced collagenⅠ/Ⅲ mRNA. It achieves this by upregulating miR-135b, which, in turn, inhibits the TGF-β/Smads pathway. This action plays a crucial role in alleviating the process of myocardial fibrosis and, as a result, hinders the progression of AF (241, 242). In summary, quercetin may serve as a potential therapeutic agent for AF by modulating myocardial fibrosis. This is achieved through its direct inhibition of oxidative stress and indirect regulation of miRNAs, inflammatory responses, and oxidative stress pathways.

#### 3.3.2 Icariin

Icariin is a flavonoid monomer extracted from Epimedium that has been proved to possess a variety of pharmacological and biological effects, including anti-inflammatory (243), activation of autophagy (244), anticancer (245), and osteoinduction effects (246). The substance has a wide range of clinical applicability, especially in the treatment of cardiovascular diseases with great potential (Figure 7) (247, 248).

Icariin can prolong the APD by inhibiting Ca^2+^ overload, thereby improving the inhomogeneity of atrial electrical conduction and directly inhibiting AER. Icariin attenuates both I_Ca, L_ and I_Na−L_ currents in rabbit cardiomyocytes. This reduces the action potential amplitude (APA) and maximum upstroke velocity, resulting in a shortened APD that helps prevent DAD and EAD (249). Ferroptosis, which can be triggered by excessive alcohol consumption, leads to the shortening of the APD and AERP through the downregulation of the protein and mRNA expression of Kv1.5, Kv2.1, Kv4.3, Cav1.2, and p-PLB, among others. Icariin activates exogenous SIRT3, which, in turn, inhibits ferroptosis and its associated processes. This action contributes to the reduction of the inducibility and duration of AF, as well as enhances the homogeneity of atrial electrical conduction (250, 251).

Icariin can also indirectly inhibit AER by affecting inflammatory responses, oxidative stress, and myocardial fibrosis. In terms of antioxidant stress, Icariin reduces the production of ROS by inhibiting the activity of Ang Ⅱ-induced NADPH oxidase (252). With regard to its anti-inflammatory properties, icariin is capable of reducing the secretion of proinflammatory factors such as IL-1β, TNF-α, iNOS, nuclear factor kappa B (NF-κB), COX-2, and the NLRP3 inflammasome. It also exerts its anti-inflammatory action by upregulating anti-inflammatory signaling pathways such as Nrf2, thereby activating the PI3 K/AKT pathway (253, 254). In the field of antimyocardial fibrosis, icariin inhibits the expression of NF-κBp65 and the TGF-β1/Smad2 signaling pathway. By improving myocardial injury, it indirectly suppresses AER (255).

#### 3.3.3 Acacetin

Acacetin is a flavonoid originally isolated from the traditional Chinese medicine *Saussurea involucrata* and found in a variety of plant and dietary sources. It has a variety of pharmacological effects, including anti-infective, anti-inflammatory, and cardioprotective effects (Figure 7) (256, 257). Acacetin inhibits the I_Kur_ and I_to_ channels, prolonging the APD and AERP in human atrial myocytes without significantly affecting the QT interval. This atrial selectivity underscores its significant therapeutic potential for AF (258). Acacetin inhibits AER mainly by inhibiting various potassium channels such as the ultrarapid delayed rectifier potassium channel (K_ur_), transient outward potassium channel (K_to_), K_ACh_, and SK (259, 260). In addition, acacetin can exert anti-inflammatory effects by inhibiting the expression of iNOS and COX-2, which, in turn, indirectly affects AER (261).

#### 3.3.4 Orientin

Orientin is a flavonoid compound with a wide range of biological activities (Figure 7), including anti-inflammatory (262), oxidative stress inhibition (263), cardioprotective (264), and antitumor (265) effects. Orientin inhibits the VDCC and ROCC to dilate blood vessels and affect AER (266, 267). In a mouse cardiac remodeling model created via left coronary artery ligation surgery, orientin effectively downregulated the expression of proinflammatory factors such as MMP-2, MMP-9, TNFα, IL-1, and IL-6. It also reduced oxidative stress by activating the eNOS/NO signaling cascade, thereby providing cardioprotective effects. This suggests that orientin may indirectly influence AER through mechanisms like anti-inflammatory action, antioxidant activity, and inhibition of myocardial fibrosis (268).

### 3.4 Polyphenols

Polyphenols are a class of complex natural compounds found in plants that contain multiple phenolic hydroxyl groups. They constitute an important component of plant secondary metabolites, encompassing not only flavonoids but also phenolic acids, quercetins, lignans, and tannins. A common characteristic of polyphenols is their potent antioxidant capacity. They exert anti-inflammatory, antifibrotic, and cardioprotective effects by scavenging free radicals and modulating enzyme activity (269).

#### 3.4.1 Curcumin

Curcumin, an extract from the rhizomes of the turmeric plant (Figure 7), possesses multiple biological activities such as anti-inflammatory, antioxidant, and antiapoptotic properties, playing a significant therapeutic role in cardiovascular diseases (270, 271).

Curcumin enhances AER by regulating ion channels in cardiomyocytes. It facilitates the uptake of intracellular Ca^2+^ into mitochondria via the uniporter pathway. Moreover, by effectively blocking the human Kv1.3 channel, curcumin inhibits the proliferation of effector memory T cells and reduces proinflammatory cytokine secretion. In addition, curcumin significantly inhibits currents such as I_Ca, L_, I_K_, and I_to_. In addition, it has a significant regulatory impact on I_Ca, L_, I_K_, and I_to_, among others (272–274).

In terms of antioxidant stress, curcumin exerts cardioprotective effects by activating the AMPK pathway through modulating uncoupling protein-2 in rats, thereby inhibiting ROS production (275). PI3K is a myocardial protective protein that plays an important role in inhibiting the pathological signaling cascade of AF. In addition, curcumin demonstrates therapeutic potential for AF by activating the Sirt1-Forkhead box-O1 (Foxo1) and PI3K-Akt signaling pathways in diabetic rats, thereby blocking Foxo1 accumulation and scavenging ROS (276). With regard to its anti-inflammatory and antifibrotic effects, curcumin has been shown to shorten the duration of Ach-CaCl_2_-induced AF in rats, inhibit left atrial fibrosis, and reduce the secretion of inflammatory factors (e.g., IL-17A, IL-1β, IL-6, and TGF-β1) in cardiomyocytes (277). It inhibits the toll-like receptor (TLR) binding mechanism of damage-associated molecular patterns, thereby significantly reducing the expression of IL-1β and IL-6 induced by NF-κB. Curcumin plays an anti-inflammatory and antifibrotic role by reducing the expression of MMP-9 and tissue inhibitor of matrix metalloproteinase-1 (TIMP-1) and alleviating the Ang Ⅱ-induced fibroblast proliferation (278, 279). Curcumin inhibits the deposition of type I and III collagen in the myocardium by activating AMPK in diabetic rats, thereby suppressing the p38 MAPK and Smad2/3 pathways (280). In summary, curcumin directly inhibits AER by suppressing the I_K_, I_Ca, L_, and I_to_ currents. Furthermore, it indirectly ameliorates AER through anti-inflammatory, antioxidant, and antifibrotic actions.

#### 3.4.2 Resveratrol

Resveratrol, a natural polyphenolic compound commonly found in grapes (Figure 7), Japanese knotweed, peanuts, and others, has been proven to possess antioxidative, anticancer, anti-inflammatory, and cardioprotective biological activities. It has unique potential in treating AF (281, 282).

The effects of resveratrol on ion channels mainly include concentration-dependent inhibition of Na_V_1.5 and inhibition of Ca^2+^ overload (283). Resveratrol upregulates SERCA2a and PLB, while downregulating NCX and CaMKII expression. By activating the PI3 K/AKT/eNOS pathway, it prolongs the APD, inhibits DAD, and prevents AF following heart failure (284, 285). In terms of antioxidative stress, resveratrol significantly reduces ROS production and counteracts oxidative stress by activating the binding of Foxo to the promoters of genes such as manganese superoxide dismutase and catalase (286, 287). With regard to its anti-inflammatory effects, resveratrol activates the Akt/mTOR pathway to downregulate factors such as IL-6 and TNF-α, thereby inhibiting endoplasmic reticulum stress. In addition, it improves mitochondrial function by inhibiting the NF-κB signaling pathway and its downstream inflammatory mediators, collectively contributing to its anti-inflammatory action (288, 289).

Resveratrol inhibits myocardial fibrosis through multiple pathways. It can alleviate excessive collagen fiber deposition by inhibiting the TGF-β/Smad3 pathway, thereby interfering with myocardial fibrosis in rats (290). In a rat model of AF induced by collagen-induced arthritis, resveratrol suppresses the overexpression of TNF-α and IL-6 in both the atria and systemically. It also inhibits apoptosis and fibrosis in rat atrial cardiomyocytes by activating AMPK and atrial Sirt1 (291, 292). In addition, resveratrol reduces the expression of MMP-2 by inhibiting NF-κB and Ang Ⅱ, which, in turn, inhibits the proliferation and differentiation of CF (293).

#### 3.4.3 Proanthocyanidins

Proanthocyanidins is a natural polyphenolic compound extracted from grape seeds and other plants (Figure 7). It has a variety of biological activities, including antioxidant, anti-inflammatory, and antitumor properties, and has many benefits for cardiovascular disease (294). In terms of improving myocardial electrical remodeling, proanthocyanidins can reduce the generation of intracellular Ca^2+^ in myocardial cells after injury. They achieve this in rat hearts by inhibiting cardiac CaMKⅡ phosphorylation, which downregulates NCX expression. This prevents intracellular Ca^2+^ overload in myocardial cells and helps maintain the ion homeostasis of cardiac cells (295). In terms of anti-inflammatory and antioxidative stress, proanthocyanidins reduce proinflammatory factors such as CRP, TNF-α, IL-6, and IL-8 by inhibiting the NF-κB/NLRP3 and MAPK signaling pathways (296, 297). In addition, they activate the PI3K/AKT signaling pathway to counter oxidative stress. This pathway stimulates the protein expression of antioxidant enzymes SOD1 and catalase, decreases ROS levels in myocardial cells, and reduces mitochondrial membrane potential (298, 299). In terms of antifibrotic, proanthocyanidins inhibit myocardial fibrosis in rats by downregulating the levels of MMP2 and TIMP2 mRNA to reduce collagen content and the expression of CTGF (300). Therefore, proanthocyanidins can help maintain the ion homeostasis of myocardial cells by inhibiting Ca^2+^ overload and play a therapeutic role in AF through their anti-inflammatory, antioxidative stress, and antimyocardial fibrosis effects.

### 3.5 Quinone compounds

Quinones are a class of natural pigments and bioactive molecules featuring a quinone structure (conjugated cyclohexadienedione). Based on their parent nucleus, they are primarily classified into benzoquinones, naphthoquinones, phenanthrenequinones, and anthraquinones. The quinone structure enables them to readily participate in redox reactions, thereby exhibiting diverse biological activities such as antibacterial, antitumor, antioxidant, and anti-inflammatory effects (301).

From an SAR perspective, the cardiac effects of quinone compounds are highly dependent on the redox properties of their quinone nucleus and substituents. Research indicates that quinones possessing “redox cycling” capabilities can generate superoxide anions through mitochondrial metabolism, indirectly mediating catecholamine release to produce positive inotropic effects. Hybrid quinones exhibiting both “redox cycling” and “alkylating” properties demonstrate enhanced potency, although their alkylating nature may carry increased toxicity risks associated with elevated atrial resting tension (302, 303).

#### 3.5.1 Tanshinone ⅡA

Tanshinone ⅡA (Tan ⅡA) is the most abundant active ingredient in Salvia miltiorrhiza (Figure 7). With pharmacological effects such as sedative, anti-inflammatory, antioxidant, and antifibrotic effects, Tan ⅡA has significant therapeutic effects on a variety of cardiovascular diseases, including AF (304–306). The effect of Tan ⅡA on ion channels is mainly related to Ca^2+^, which can directly inhibit the influx of Ca^2+^ in vascular smooth muscle cells. In the early stage of myocardial ischemia, Tan ⅡA exerts a protective effect on cardiomyocytes by inhibiting intracellular Ca^2+^ and cell adhesion pathways (307, 308). In a rabbit model of chronic heart failure induced by rapid ventricular pacing, Tan ⅡA significantly prolonged the AERP, moderately increased the conduction time in the atrium, and reduced the inductivity of AF by improving AER (309).

In addition, Tan ⅡA can also indirectly affect atrial remodeling by inhibiting myocardial fibrosis, reducing oxidative stress, and decreasing anti-inflammatory effects. Tan ⅡA inhibits the progression of myocardial fibrosis by inhibiting NOX2 and Ang Ⅱ/TGF-β1/Smad2/3 signaling. In addition, Tan ⅡA possesses antioxidative stress properties that can mitigate oxidative stress induced by NOX2 and Ang Ⅱ. This, in turn, indirectly impacts AER (310, 311). In addition, Tan IIA activates the SIRT1/PGC1α pathway to maintain mitochondrial energy metabolism in animal cells and mitigate oxidative stress damage (312). With regard to its anti-inflammatory properties, Tan IIA plays a significant role in downregulating the mRNA expression of inflammatory factors in human U87 glioblastoma cells such as IL-1β, TNF-α, and IL-6. It achieves this by inhibiting the TLR4/NF-κB/MAPK signaling pathway, highlighting its crucial role in anti-inflammatory processes (313).

#### 3.5.2 Barbaloin

Barbaloin is the main active ingredient in Aloe vera and possesses pharmacological effects such as anti-inflammatory, antibacterial, and cardiomyocyte protective properties (Figure 7) (314–316). Cao et al. (317) used whole-cell patch clamp techniques to record ion currents in isolated rabbit ventricular cardiomyocytes. The results demonstrated that barbaloin concentration-dependently inhibited peak I_Na_ and I_Ca, L_. It reversibly inhibited ATXⅡ-induced I_Na−L_, thereby eliminating ATXⅡ-induced EAD and Ca^2+^-induced DAD, while prolonging the APD. Therefore, barbaloin primarily improves AER by directly inhibiting I_Na−L_ and Ca^2+^ overload, thus playing a therapeutic role in AF.

### 3.6 Terpenoid compounds

Terpenoid compounds, also known as terpenoids, constitute a large family of natural products built from isoprene (C5H8) units. Based on the number of isoprene units, they can be classified into monoterpenes (C10), sesquiterpenes (C15), diterpenes (C20), triterpenes (C30), and tetraterpenes (C40). They are widely present in numerous important pharmaceuticals and exhibit pharmacological activities such as anti-inflammatory, antioxidant, and cardiovascular protective effects (318).

#### 3.6.1 Paeoniflorin

Paeoniflorin is a water-soluble monoterpenoid glycoside extracted from various plants such as red peony, white peony, and tree peony (Figure 7). It possesses pharmacological effects such as anti-inflammatory, immunomodulatory, antitumor, and protection against cardiac I/R injury (319–321). Paeoniflorin primarily impacts AER by inhibiting I_Na_ and I_K1_, as well as suppressing the Ca^2+^/CaMKⅡ signaling pathway (322, 323). In a study conducted by Tsai et al. (324) using an *in vitro* rat model of lycorine-induced arrhythmias, it was observed that the antiarrhythmic effect of paeoniflorin remained unaffected by tetrodotoxin and quinidine. This indicates that the ability of paeoniflorin to inhibit lycorine is primarily associated with the inhibition of intracellular Ca^2+^ influx. In addition, PF reduced the risk of AF in mice by inhibiting the PI3K-Akt pathway and attenuating Ang II-induced myocardial fibrosis (325).

### 3.7 Chinese herbal compounds

Chinese herbal compounds are compound preparations created by combining multiple herbal ingredients based on TCM theory and clinical experience. Their therapeutic effects are grounded in the “sovereign, minister, assistant, and messenger” principle of formulation. Through the synergistic action of multiple active components (such as alkaloids, saponins, and flavonoids mentioned earlier), these formulas achieve integrated regulation across multiple targets and pathways, aiming to restore the body's equilibrium. Modern research focuses on elucidating the scientific relationship between their complex chemical constituents and their overall pharmacological effects.

#### 3.7.1 Shensong Yangxin capsule

Shensong-Yangxin capsule (SSYX) capsule is a traditional Chinese medicine composed of herbs such as Ren Shen, Mai Dong, Wu Wei Zi, Sang Ji Sheng, Shan Zhu Yu, Suan Zao Ren, Gan Song, Dan Shen, and Huang Lian. Numerous drugs incorporate a substantial amount of active ingredients, mainly including ginsenosides (326), lignans (327), ziziphus jujuba saponins (328), tanshinones (329), paeoniflorin (330), and berberine (331). They collectively exert anti-inflammatory, antiapoptotic, antioxidative, immunoregulatory, and cardiovascular protective effects. SSYX is widely recognized for its efficacy in treating cardiovascular diseases, as it helps regulate myocardial energy metabolism, inhibits cardiomyocyte hypertrophy and apoptosis, and enhances myocardial blood flow (332–334). Clinical studies have highlighted that SSYX therapy offers a more effective and safer alternative to traditional treatments for paroxysmal AF. Researchers have been delving into its underlying mechanisms with notable findings (335, 336). In the realm of ventricular remodeling, SSYX markedly increases the expression of Kv4.2, Kv4.3, CaV1.2, and Cx43, while reducing the densities of I_to_ and I_K1_. SSYX effectively alleviates Ca^2+^ overload by downregulating the TLR4/MyD88/CaMKII signaling pathways. This significantly prolongs the APD and shortens DAD in rats (337, 338). Therefore, it is hypothesized that SSYX also has a relevant effect on AER, and the specific mechanism needs to be further studied. Addressing antimyocardial fibrosis, SSYX elevates ferroportin levels, which, in turn, inhibits the expression of collagen-I, collagen-Ⅲ, and TGF-β mRNA. This mechanism reduces the susceptibility to AF by curbing atrial fibrosis in rats (339). In addition, SSYX decreases the expression of MMP-9 and TIMP-1, which further inhibits atrial fibrosis in rats, enhances atrial conduction function, and prevents AF postheart failure (340). In terms of its anti-inflammatory effects and ANS regulation, SSYX therapy counters SN overactivation and lowers levels of inflammatory markers such as TNF-α and IL-6, particularly in the context of long-term intermittent atrial pacing. Moreover, it hampers the progression of AF by modulating cholinergic anti-inflammatory pathways (341).

In conclusion, SSYX exerts a direct influence on the AERP by mitigating Ca^2+^ overload, diminishing the densities of I_to_ and I_K1_ currents, and extending the APD. Beyond these direct effects, SSYX also indirectly impacts AER through a variety of mechanisms, including its anti-inflammatory properties, antimyocardial fibrosis capabilities, and modulation of the ANS. Collectively, these actions contribute to cardiac protection, underscoring the promising potential of SSYX in clinical applications.

#### 3.7.2 Wenxin Keli

Wenxin Keli (WXKL) primarily consists of Codonopsis, Panax notoginseng, Gansong, amber, and Polygonatum as the main components. The formulation includes multiple active ingredients such as ginsenosides (326), notoginsenosides (342), and succinic acid (343). It synergistically enhances coronary artery blood flow, reduces myocardial oxygen consumption, and alleviates cardiac preload and afterload. WXKL has demonstrated effectiveness in shortening QRS and QT intervals, showing promising efficacy in treating various cardiovascular conditions, including chronic heart failure, arrhythmias, and cardiac hypertrophy (344). One of the key mechanisms by which WXKL treats AF is through direct modulation of ion channels. It inhibits I_to_ and exhibits a preferential inhibitory effect on I_Na_ in atria. In addition, WXKL suppresses I_Ca, L_ by downregulating both the protein level and the phosphorylation of CaMKⅡ, which, in turn, reduces EAD and DAD and shortens the APD (345–347). WXKL also addresses oxidative stress by diminishing the overproduction of ROS through the inhibition of the PKC-*δ*/NOX2 pathway. Moreover, it improves atrial remodeling by enhancing mitochondrial function, thereby offering cardioprotective effects (348, 349). In terms of combating myocardial fibrosis, WXKL alleviates cardiac fibrosis and improves cardiac function in rats, an effect mediated by suppressing structural remodeling via reduced collagen-I levels (350). In summary, the multifaceted approach of WXKL includes directly targeting mechanisms responsible for Ca^2+^ overload, I_to_, and I_Na_, which inhibits the AERP. Its potential as a therapeutic option for AF is further bolstered by its capacity to inhibit ROS generation and reduce myocardial fibrosis, making it a promising candidate in the field of cardiovascular treatment.

#### 3.7.3 Xin–Su–Ning capsule

**Figure 8:**
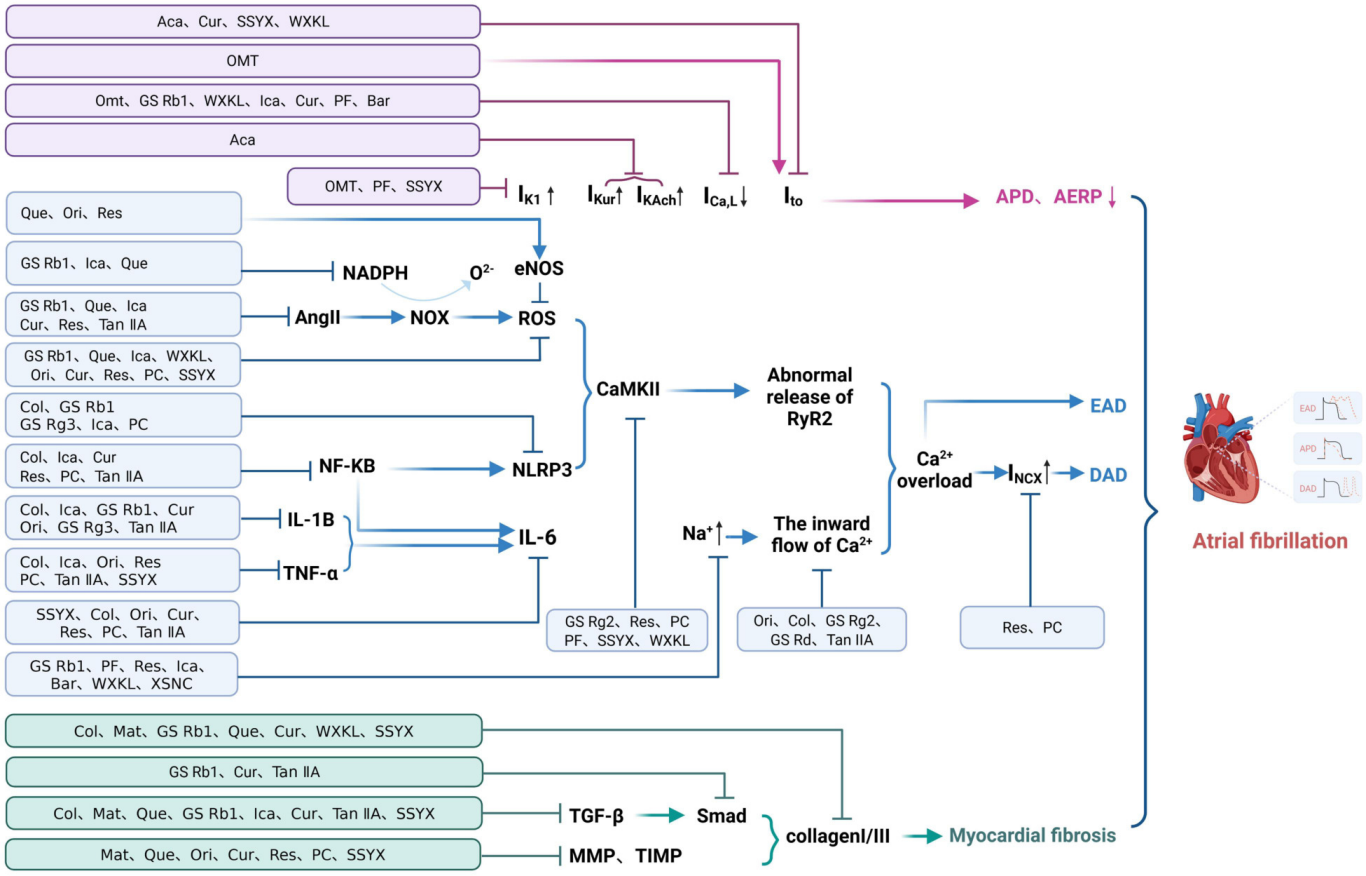
The mechanism of natural drugs and active components in treating atrial fibrillation through the regulation of atrial electrical remodeling. Natural drugs and their active ingredients primarily enhance myocardial electrical remodeling by regulating ion homeostasis, inflammatory responses, oxidative stress, and myocardial fibrosis mechanisms. With the rise of disciplines such as natural pharmacology and phytochemistry, clinicians and researchers have increasingly focused on the study and review of natural drugs and active ingredients. By Figdraw (http://www.figdraw.com).

Xin–Su–Ning capsule (XSNC) is a distinguished proprietary Chinese medicine consisting of a blend of traditional ingredients, including Huang Lian, Ban Xia, Fu Ling, Zhi Shi, Chang Shan, Lian Zi Xin, Ku Shen, Qing Hao, Ren Shen, Mai Dong, and Gan Cao. It has gained widespread recognition in treating cardiac arrhythmias. Active ingredients such as berberine (331), Poria cocos polysaccharides (351), liensinine (352), sophoridine (214), and artemisinin (353) constitute the material foundation for its therapeutic efficacy. Researchers have constructed a drug target–disease–gene interaction network and found that the hubs of the XSNC's hypothetical target-arrhythmia-related gene interaction network are significantly involved in signaling pathways such as VEGF, G protein-coupled Ach receptors, and αAR. This suggests that XSNC may help repair ischemic cardiomyocytes and reverse the imbalance in the sympathovagal system during arrhythmias (354). A network pharmacology study has revealed that XSNC effectively balances ion flow in cardiomyocytes through the regulation of various ion channels. This action not only protects the heart from I/R injury but also plays a crucial role in inhibiting cardiomyocyte apoptosis and bolstering energy metabolism within heart cells (355). Mechanistic studies have shown that XSNC can treat premature ventricular contractions by inhibiting APA and prolonging APD through the blockade of hNa_V_1.5 and human ether-a-go-go-related gene (hERG) channels in rats (356). Based on these findings, we hypothesize that XSNC also exerts beneficial effects on AER. In conclusion, the direct influence of XSNC on AER is primarily achieved by modulating sodium and potassium channels and extending the APD. In addition, its indirect effects on AER include the regulation of the ANS and the improvement of energy metabolism in cardiomyocytes. This multifaceted approach underscores the potential of XSNC as an effective treatment for cardiac arrhythmias (Table 1) (Figure 8).

**Table 1:** Summary of the mechanisms of natural drugs and active ingredients in the treatment of AF.

Natural drugs and active ingredients	Specific ingredients	Disease	Model category	Model	Drug dosage	Targeted molecules or targeted pathways	PMID
Alkaloids	Colchicine	AF	*In vitro* experiment	HL-1 cells derived from mouse atrial cardiac muscle cells	3 nM	Inhibits the phosphorylation of calcium channels	203
		AF	*In vivo* experiment	Rat sterile pericarditis model induced by the epicardial application of sterile talc	0.5 mg kg^−1^·day^−1^	Significantly inhibits the phosphorylation of signaling molecules such as p38 MAPK, AKT, JNK, and NF-κB	204
		Myocardial fibrosis	*In vivo* experiment	AF rat model induced by Ach-CaCl_2_	0.8 mg/kg	Suppression of TGF-β1, collagen I, and collagen Ⅲ synthesis	205
		Cardiac remodeling	*In vivo* experiment	TAC mice	–	Activate the SIRT3 protein	206
	Matrine	AF	*In vivo* experiment	AF rat model induced by electric pacing	15 、 30 、45 mg/kg	Increased α1C/Cav1.2 and I_Ca, L_ expression and decreased M3-R and IKM3 expression	208
		AF	*In vivo* experiment	TAC mice	50 、100 、10 mg/kg	inhibit Wnt3a/β-catenin the signaling pathway	210
		MI	*In vivo* experiment	MI rat model induced by a ligation of the left anterior descending coronary artery	–	Reduce the expression of TGF-β1 and MMP-9 by lowering the levels of type I and Ⅲ collagen	211
	Oxymatrine	Arrhythmic	*In vivo* experiment	Arrhythmic rat model induced by coronary ligation	–	Reduce I_Ca, L_ and I_K1_ and enhance I_to_	216
	Berberine	Myocardial fibrosis	*In vivo* experiment	Arrhythmic rat model induced by RAP	–	Activating the (AMPK)-PPARα signaling pathway	220
		AF	*In vivo* experiment	AF rabbit model induced by ACh	2 、1 mg/kg	Inhibit potassium channels and stimulate Na ^+^ - Ca^2+^ exchange	221
Saponins	Ginsenoside Rb1	AF	*In vitro* experiment	Isolated rabbit ventricular myocytes	1、5、10、20 μmol/L	Inhibit I_Na_ and I_Ca, L_ and RyR2 protein levels and upregulate SERCA protein levels	225
		Cardiotoxicity	*In vivo* experiment	Aconitine-induced rat cardiotoxicity	10、20、40 mg/kg	Lower levels of caspase-1, NLRP3, IL-1β, and IL-18	226
		I/R injury	*In vivo* experiment	Mice subjected to I/R injury	50 mg/kg	Curtail mitochondrial ROS production and NADH dehydrogenase activity	227
		HF	*In vivo* experiment	HF rat model induced by abdominal aortic coarctation	35、70 mg/kg	Inhibit the TGF-β1/Smad pathway, downregulate type I collagen, Ang Ⅱ, ACE, and AT1 receptors	228
	Ginsenoside Re	EMA	*In vivo* experiment	EMA cat cardiomyocytes induced by electrically pacing cardiomyocytes	–	Enhance the frequency of RyR channel openings	224
	Ginsenoside Rg2	HF	*In vivo* experiment	Chloride-induced arrhythmia mice	10 nM	Inhibit the phosphorylation of CaMKII*δ*	230
	Ginsenoside Rg3	–	*In vivo* experiment	Mouse macrophage	–	Inhibit lipopolysaccharides induction and the activation of the NLRP3 inflammasome and IL-1β	232
	Ginsenoside Rd	–	*In vivo* experiment	Fresh isolated rat aorta smooth muscle cells	–	Inhibit the activation of α1AR and reduce Ca^2+^ inward flow	233
Flavonoid compounds	Quercetin	Spontaneously hypertensive	*In vivo* experiment	Spontaneously hypertensive rat model induced by acetylcholine	10 mg/kg	Reduce the activity of NADPH oxidase and the content of ROS	238
		Spontaneously hypertensive	*In vivo* experiment	Spontaneously hypertensive rats	20 mg/kg/day	Modulate the SIRT3/PARP-1 pathway	239
		Hypertension	*In vivo* experiment	Sprague–Dawley two-kidney, one-clip rats	10 mg/kg/day	Decrease TGF-β and MMP activity	240
		Myocardial fibrosis	*In vivo* experiment	Cardiac remodel mice induced by infusion of Ang II	25 mM/kg	Inhibit the expression of Collagen I and Collagen III	241
		AF	*In vivo* experiment	Isoprenaline-induced AF rats	25 mg/kg/day	Inhibit the TGF-β/Smads pathway via promoting miR-135b expression	242
	Icariin	Arrhythmias	*In vivo* experiment	Aconitine-induced arrhythmias rabbits	3 mg/kg	Attenuate both I_Ca, L_ and I_Na−L_ currents	249
		AF	*In vivo* experiment	Excessive alcohol-treated C57BL/6J mice	50 mg/kg/day	Activating SIRT3-AMPK signaling	250
		Hypertension	*In vivo* experiment	AngII-induced hypertension rat model	10 mg/kg/day	Inhibit the activity of Ang Ⅱ-induced NADPH oxidase	252
		Cellular inflammation	*In vivo* experiment	Inflammation rats induced by carrageenan	50 mg/kg	Increase both Nrf2 and HO-1 expression of mRNA	254
		Myocardial fibrosis	*In vivo* experiment	Spontaneously hypertensive rats	4、8、16 mg/kg	Inhibit the expression of NF-kB signaling and the TGF-β1/Smad2 signaling pathway	255
	Acacetin	AF	*In vitro* experiment	Human atrial myocytes and guinea pig cardiac myocytes	5 mg/kg	Inhibit I_kur_ and I_to_ channels	258
		J wave syndromes	Clinical trial	Kv4.3-V392I iPSC-CMs	10、30 μM	Inhibit I_to_	259
		AF	*In vitro* experiment	Establish the HEK 293 cell lines stably expressing the SK_Ca_1, SK_Ca_2, and SK_Ca_3 channels	10 μM	Inhibit various potassium channels such as K_ur_, K_to_, K_ACh_, and SK	260
		Inflammation	*In vitro* experiment	Lipopolysaccharide (LPS)-induced murine macrophages	10 μM	Inhibit the expression of iNOS and COX-2	261
	Orientin	–	*In vitro* experiment	Isolated thoracic aortic rings from New Zealand rabbit	–	Inhibition of both intracellular Ca^2+^ release and extracellular Ca^2+^ influx	266
		–	*In vitro* experiment	Rat endothelial cell cultures	3、10、30、and 100 µM	Calcium mobilization	267
		Myocardial infarction	*In vivo* experiment	Mice cardiac remodeling model established by left coronary artery ligation surgery	40 mg/kg	Downregulate the expression of MMP-2, MMP-9, TNFα, IL-1, and IL-6 and activate the eNOS/NO signaling cascade	268
Polyphenols	Curcumin	–	*In vitro* experiment	Rat hippocampal neurons	1 μM	Selectively inhibits L-type Ca^2+^ channel currents	272
		Autoimmune diseases	*In vitro* experiment	HEK-293 cells and effector memory T cells isolated from patients	–	Inhibit the hKv1.3 channel	273
		Apoptosis	*In vitro* experiment	Human leukemia U937 cells	–	As a stimulator of intracellular Ca^2+^ uptake into mitochondria	274
		Age-related cerebrovascular dysfunction	*In vivo* experiment	Male Sprague–Dawley (SD) rats and UCP2 knockout (UCP2−/−) and matched wild-type mice	0.2%, 10 μmol/L	Regulate uncoupling protein-2 to activate the AMPK pathway	275
		Diabetic cardiomyopathy	*In vivo* experiment	Streptozoticin-induced diabetic rats	–	Activate the Sirt1-Foxo1 and PI3K-Akt signaling pathways	276
		AF	*In vivo* experiment	AF rat model induced by Ach - CaCl2	4 mL/kg, concentration: 50 mg/mL	Reduce the secretion of IL-17A, IL-1β, IL -6, and TGF-β1	277
		Cardiac fibrosis	*In vivo* experiment	Cardiac fibrosis rats induced by ISO	150 or 300 mg/kg/day	Decrease TGF-β1, MMP-9, and TIMP-1 expression and reduce the protein expression of collagen type I/III in hearts	278
		Cardiac fibrosis	*In vivo* experiment	Experimental diabetes rats induced by the injection of low-dose streptozotocin (STZ) and high-energy diet	25 μmol/L	Inhibit TGF-β1 production and canonical Smad signaling and block the non-canonical AMPK/p38 MAPK pathway	280
	Resveratrol	AF	*In vitro* experiment	tsA201 cells, human atrial samples, and rat myocytes	0.3、1 mg/kg	Concentration-dependent inhibition of NaV1.5 and inhibition of Ca^2+^ overload	283
		AF	*In vivo* experiment	HF rabbits induced by undergoing coronary ligation	–	Activate the PI3 K/AKT/eNOS signaling pathway	284
		Cardiac hypertrophy	*In vivo* experiment	Cardiac hypertrophy males induced by the aortic banding procedure	4 mg/kg/day	Reduce the expression of NCX and CAMKⅡ	285
		Oxidative stress	*In vitro* experiment	Primary human or porcine lens epithelial cells incubated under chronic hyperoxic (40%) oxygen conditions	25 μM	Inhibition of FoxO1A, FoxO3A, and FoxO4	286
		Nephrotoxicity	*In vitro* experiment	Mouse renal tubular epithelial (TCMK-1) cells exposed to Cd	–	Activate the Sirt3/FoxO3a signaling pathway	287
		Oxidative stress	*In vitro* experiment	Acrylamide-induced oxidative stress in HepG2 liver cells	–	Inhibit the NF-κB signaling pathway and its downstream inflammatory mediators	288
		Inflammation	*In vivo* experiment	Allergic inflammation mice induced by aspergillus fumigatus	–	Activate the Akt/mTOR pathway	289
		Myocardial fibrosis	*In vitro* experiment	Neonatal rat CFs treated with TGF-β1	12.5、25、50、75and 100 μM	Inhibit the TGF-β/Smad3 pathway	290
		AF	*In vivo* experiment	collagen-induced arthritis rats	10 mg/kg/day	Suppress TNF-α and IL-6 and activate AMPK	291
		–	*In vitro* experiment	Isolated adult rat CF	5–25 microM	Inhibit NF-κB and Ang Ⅱ and reduce the expression of MMP-2	293
	Proanthocyanidins	Hypertension and heart failure	*In vivo* experiment	Aldosterone-treated rats	5 mg/kg/day	Inhibit the self-phosphorylation of cardiac CaMKⅡ	295
		Gestational diabetes mellitus (GDM)	*In vivo* experiment	GDM mice model built by feeding a high-fat-high-sucrose diet	27.8 mg/kg/day	Inhibit the NF-κB/NLRP3 pathway	296
		Inflammation	*In vitro* experiment	LPS-stimulated RAW264.7 cells	0, 20, 40, 80 μM	Reduce proinflammatory factors such as CRP, TNF-α, IL-6, and IL-8	297
		Spinal cord injury (SCI)	*In vitro* experiment	PC12 cells with oxidative damage induced by H_2_O_2_	5、15、25 μM	Activate the PI3K/AKT signaling pathway	299
		Cardiac alterations	*In vivo* experiment	Aldosterone-salt-treated rats	100 mg/kg/day	Downregulate the levels of MMP2 and TIMP2 mRNA	300
Quinone compounds	Tanshinone ⅡA	MI	*In vivo* experiment	MI rats induced by the occlusion of the left anterior descending coronary artery	–	Inhibit intracellular Ca^2+^ and cell adhesion pathways	307
		–	*In vitro* experiment	Rat-isolated coronary artery	100 µM	Inhibit the influx of Ca^2+^ in vascular smooth muscle cells	308
		Myocardial fibrosis	*In vivo* experiment	LPS-induced cardiac fibrosis mice	–	Inhibit NOX2	310
		Atrial fibrosis	*In vitro* experiment	Human atrial fibroblasts stimulated with Ang II	0、5、25、50、100, and 200 μM	Inhibit Ang Ⅱ/TGF-β1/Smad2/3 signaling	311
		IR injury	*In vivo* experiment	IR mice induced by the left anterior descending coronary artery with a knot	5 and 25 mg/kg	Activate the SIRT1/PGC1α pathway	312
		Neuroinflammation	*In vitro* experiment	LPS-induced neuroinflammation and neurotoxicity in human U87 astrocytoma cells	1、5、10、20, and 40 μM	Inhibit the TLR4/NF-κB/MAPK signaling pathway	313
	Barbaloin	Ventricular arrhythmias	*In vitro* experiment	Isolated rabbit ventricular myocytes	100 and 200 μmol/L	Inhibit peak sodium current, I_Na−L,_ and I_Ca, L_	317
Terpenoid compounds	Paeoniflorin	–	*In vitro* experiment	Rat ventricular myocytes	100 μmol/L	Block I_Ca, L_, I_Na_, and I_K1_ without affecting I_to_, I_Ks_, or I_Kr_	322
		Neurodegenerative diseases	*In vitro* experiment	Glutamate (Glu)-induced PC12 cell damage	50–200 μM	Prevent intracellular Ca^2+^ overload and suppress the overexpression of CaMKII	323
Chinese herbal compounds	SSYX	Ischemic arrhythmia	*In vivo* experiment	Ischemic arrhythmia rat cardiomyocytes induced by myocardial ischemia	1.8 g/kg	Increase the expression of Kv4.2, Kv4.3, CaV1.2, and Cx43, reduce the densities of Ito and IK1, and inhibit the TLR4/MyD88/CaMKII signaling pathway	338
		AF	*In vivo* experiment	MS rats induced by high-carbohydrate, high-fat diet together with 25% fructose in drinking water	0.8、0.4 g/kg	Elevate ferroportin levels and inhibit the expression of collagen-I, collagen-Ⅲ, and TGF-β mRNA	339
		AF	*In vivo* experiment	MI induction rat model	600 mg/kg	Downregulate TGF-β1, MMP-9, TIMP-I, and type I and III collagen expressions	340
		Paroxysmal AF	*In vivo* experiment	Paroxysmal AF dog model induced by long-term intermittent atrial pacing	–	Reduce inflammatory markers such as TNF-α and IL-6 and inhibit the decrease in Ach	341
	WXKL	Ischemic stroke-induced AF	*In vivo* experiment	Ischemic stroke-induced AF rats induced by cerebral artery occlusion/reperfusion	1.35、4.05、8.1 g/kg	Regulate the cholinergic-calcium signaling pathway	345
		Ventricular arrhythmias	*In vivo* experiment	Ischemia-induced ventricular arrhythmias rats	8 g/kg	Inhibit I_CaL_ and I_to_	346
		AF	*In vitro* experiment	Canine atrial and ventricular myocytes	5、10 g/L	Atrial selectivity to block I_Na_	347
		I/R injury	*In vitro* experiment	H9c2 cardiomyocyte cell line subject to hypoxia/reoxygenation (H/R)	5 mg/mL	Inhibit PKC-*δ*/NOX2/ROS signaling	348
		Mitochondrial dysfunction	*In vitro* experiment	Rat model of type 2 diabetes induced by high-fat feeding and low STZ injection	1、3 g/L	Regulate the activation of signaling pathways induced by H_₂_O_₂_ and reduce ROS	349
		Cardiac hypertrophy and arrhythmias	*In vivo* experiment	TAC rats underwent transverse aortic constriction surgery	4 g/kg/day	Regulate the CaMK II signaling pathway and reduce type III collagen levels	350
	XSNC	Arrhythmias	*In vitro* experiment	Isolated rat ventricular myocytes	0.4 g/L	Block hNaV1.5 and hERG channels	356

## 4 Deficiencies and limitations

In conclusion, natural drugs and their active ingredients hold significant potential in modulating the mechanisms of AER. However, considering the complexity of the mechanism of AER and the diversity of natural drugs and their active ingredients, there are inherent challenges and limitations in advancing related research: (1) Research into the roles of miRNA expression and ANS remodeling in myocardial electrical remodeling remains nascent. In addition, the exploration of the intricate interrelationships among the diverse factors contributing to AF has not yet been thoroughly conducted. (2) Both downregulation and upregulation of I_Ca, L_ are different causes of AF, demonstrating the importance of cardiomyocyte ion homeostasis in maintaining normal cardiac rhythm. Therefore, there is a need for more in-depth research into the homeostatic regulation of different ions and their interrelated dynamics. (3) In clinical research on TCM formulas treating AF, the formulations feature multicomponent, multitarget, and multipathway advantages, often holding substantial clinical importance. However, the complexity of components in TCM formulations increases research challenges, and their quality and dosage ratios affect the therapeutic outcomes; thus, it 's essential to systematically advance research from active ingredients to individual drugs to combinations to fully uncover the mechanisms of TCM effectiveness. (4) Clinical research on TCM for AF is often limited by small sample sizes, unclear blinding methods, high heterogeneity, and risk of bias. These methodological limitations significantly hinder the advancement of robust clinical research on TCM. Therefore, establishing robust clinical evaluation systems, such as Core Outcome Sets, is crucial for advancing the field. (5) Although natural compounds have demonstrated promising therapeutic outcomes in AF treatment, they are currently used primarily as adjunctive, alternative, or combination therapies in clinical practice. (6) Mechanistic studies predominantly rely on acute or short-term animal models of AF, whose pathological progression and electrical remodeling characteristics differ fundamentally from those of chronic persistent AF in humans, limiting the applicability of research findings to chronic clinical patients. (7) While existing studies have revealed the regulatory effects of natural compounds on electrophysiological parameters or molecular pathways, the association between these experimental findings and clinical hard endpoints—such as stroke incidence, heart failure hospitalization, or improved survival rates—remains unestablished.

While the majority of natural drugs and active ingredients exhibit antiarrhythmic benefits, some of them have been associated with arrhythmogenic effects in clinical and basic research. Reports indicate that colchicine consumption has been associated with three cases of EMD cardiac arrest and one self-limiting ventricular tachycardia, suggesting potential myocardial and conduction system toxicity. Excessive doses may induce arrhythmias and hemodynamic instability, effects mediated through interactions with cardiomyocyte tubulin (357). Matrine and berberine can concentration-dependently inhibit hERG potassium channels, leading to QT interval prolongation and increased risk of arrhythmia (358–360). Clinical overdose may induce serious adverse reactions such as bradycardia and atrioventricular block. Ginsenoside Rg3 has been found to disrupt vascular smooth muscle function and lead to weakened vasoconstriction and structural remodeling by blocking Ca2+ influx via the LTCC (361). In addition, studies have shown that both synephrine and artemisinin also pose risks of inducing arrhythmias (362, 363). Consequently, clinicians must be aware of the bidirectional effects and potential risks associated with using natural drugs and active ingredients, carefully determining dosages and thoroughly evaluating the safety of combination therapies. Researchers must also prioritize the meticulousness of their research methodologies to facilitate the judicious use of natural drugs.

## 5 Discussion

The clinical outcomes of AF are complex, and timely interruption of the disease progression and symptoms is crucial. Atrial electrical and structural remodeling form crucial foundations for the initiation and persistence of AF. Prolonged myocardial electrical disturbances can induce alterations in the atrial structure, impacting myocardial electrophysiological activity. These interconnected processes involve miRNA expression, inflammatory responses, oxidative stress, and ANS remodeling, forming a complex network. Pharmacological studies have focused on the effects of natural drugs and active components on specific ion channels to alleviate arrhythmias (364, 365). In terms of AF mechanisms, a significant number of studies have explored its electrophysiological and structural remodeling mechanisms (366–368), but there has yet to be a comprehensive and detailed summary that connects natural drugs and active components with these mechanisms. This review thus explores the fundamental mechanisms underlying AF induction, offering a comprehensive review of AER mechanisms and current research on natural drugs and their active ingredients. It aims to provide a broad perspective and reference for clinical practitioners and researchers involved in precise drug development and design.

As research technologies advance and investigations deepen, the mechanisms underlying AER in AF will become increasingly elucidated. This progress promises to offer a wider array of research and treatment approaches for scientists, technologists, and clinical researchers. Currently, some natural drugs and active ingredients such as Salvia miltiorrhiza injection and ginseng stem and leaf saponin capsules are already in widespread clinical use. Research into the mechanisms of natural pharmaceutical active ingredients and the introduction of new drugs to the market are progressively being undertaken. The therapeutic efficacy and targets of these natural drugs and their active ingredients are poised for broader exploration and application. This burgeoning field is anticipated to evolve into a new domain for AF treatment, characterized by personalized diagnosis and treatment systems. In addition, this new domain offers enhanced prospects and ideas for researchers specializing in precision drug development and design. It is firmly believed that with the concerted efforts of researchers and clinicians, the development and application prospects of natural drugs targeting AER for the treatment of AF will become even broader and more promising.

## 6 Conclusion

In summary, natural drugs and their active ingredients primarily ameliorate myocardial electrical remodeling by regulating ion homeostasis, inflammatory responses, oxidative stress, and myocardial fibrosis mechanisms. In addition, specific compounds exhibit unique mechanisms: resveratrol may exert anti-inflammatory and anti-AER effects via improved mitochondrial function; icariin may suppress ferroptosis-induced electrophysiological disturbances; and acacetin may prevent AF through selective inhibition of atrial potassium currents. These active ingredients with unique mechanisms of regulating AER may provide ideas for future treatment of AF and development of new drugs through further studies. With the rise of disciplines such as natural pharmacology and phytochemistry, clinicians and researchers have increasingly focused on the study and review of natural drugs and active ingredients.

## Glossary

Ach: acetylcholine
AER: atrial electrical remodeling
AERP: atrial effective refractory period
AERPD: atrial effective refractory period dispersion
AF: atrial fibrillation
AMPK: AMP-activated protein kinase
Ang Ⅱ: angiotonin Ⅱ
ANS: autonomic nervous system
APA: action potential amplitude
APD: action potential duration
BCL-2: B-cell lymphoma-2
Ber: berberine
BRD4: bromodomain-containing protein 4
Ca^2+^: calcium
CACNA1C: calcium voltage-gated channel subunit alpha 1c
CACNB1: calcium voltage-gated channel auxiliary subunit beta
CaMKII: Ca^2+^/calmodulin-dependent protein kinase II
CaT: calcium transient
CF: cardiac fibroblasts
CRP: C-reactive protein
CTGF: connective tissue growth factor
Cx40: connexin 40
Cx43: connexin 43
DA: dopamine
DAD: delayed after depolarization
DVL2: dishevelled 2
EAD: early after depolarization
EMA: electromechanical alternans
EPI: epinephrine
FGF-21: fibroblast growth factor 21
hERG: human ether-a-go-go-related gene
hs-CRP: high-sensitivity C-reactive protein
I/R: ischemia/reperfusion
I_Ca,L_: L-type calcium current
I_K_: delayed rectifier potassium current
I_K1_: inward rectifier potassium current
I_KACh_: acetylcholine-activated inwardly rectifying potassium current
I_K−ATP_: ATP-sensitive potassium current
IKM3: the M3 receptor-mediated K^+^ current
I_Kr_: rapid delayed rectifier potassium current
I_Ks_: slow delayed rectifier potassium current
I_Kur_: ultrarapid delayed rectifier potassium current
IL: interleukin
I_Na_: sodium current
I_Na−L_: late sodium current
I_NCX_: Na^+^-Ca^2+^ exchange current
IP3: inositol 1,4,5-trisphosphate
IP3R: inositol 1,4,5-triphosphate receptor
ISO: isoprenaline
I_to_: transient outward potassium current
JNK: c-Jun N-terminal kinase
K^+^: potassium
K_2P_: two-pore-domain potassium channel
K_ACh_: acetylcholine-activated inwardly rectifying potassium channel
K_ATP_: ATP-sensitive potassium channel
K_Ca3.1_: the intermediate-conductance Ca^2+^-activated K^+^
K_to_: transient outward potassium channel
K_ur_: ultrarapid delayed rectifier potassium channel
LGCC: ligand-gated calcium channel
LQT: long QT syndrome
LTCC: L-type calcium channel
M3-R: M3 receptor
MAPK: mitogen-activated protein kinase
Mfn1: mitofusin1
MMP: matrix metallo proteinase
Na^+^: sodium
NADPH: nicotinamide adenine dinucleotide phosphate
NCX: Na^+^-Ca^2+^ exchanger
NE: norepinephrine
NFAT: nuclear factor of activated T cells
NF-κB: nuclear Factor kappa B
NLRP3: NACHT, LRR, and PYD domain-containing protein-3
nNOS: neuronal nitric oxide synthase
NO: nitric oxide
NOS: nitric oxide synthase
NOX: nicotinamide adenine dinucleotide phosphate oxidase
NRF-1: nuclear respiratory factor-1
PARP-1: poly-ADP-ribose polymerase-1
Pd: P-wave dispersion
PDGF: platelet-derived growth factor
P13K: phosphoinositide 3-kinase
PKC: protein kinase C
PLC-PKC: phospholipase C-protein kinase C
PNS: parasympathetic nervous system
Rac1: Ras-related C3 botulinum toxin substrate 1
RAS: renin–angiotensin system
ROCC: receptor-operated calcium channel
ROS: reactive oxygen species
RyR: ryanodine receptor
RyR2: ryanodine receptor 2
SERCA: sarco/endoplasmic reticulum Ca^2+^-ATPase
SIRT3: sirtuin3
SK: small-conductance calcium-activated potassium channel
SN: sympathetic nervous
SNS: sympathetic nervous system
SOCC: store-operated calcium channel
SR: sarcoplasmic reticulum
SSYX: Shensong–Yangxin capsule
STAT3: signal transducer and activator of transcription 3
Tan ⅡA: tanshinone ⅡA
TFAM: mitochondrial transcription factor A
TIMP-1: tissue inhibitor of matrix metalloproteinase-1
TNF-α: tumor necrosis factor-alpha
TRPC3: transient receptor potential canonical-3
TTCC: T-type calcium channel
VDCC: voltage-dependent calcium channel
VN: vagus nerve
WXKL: Wenxin Keli
XO: xanthine oxidase
XOR: xanthine oxidoreductase
XSNC: Xin–Su–Ning capsule.
ZFHX3-KD: zinc finger home obox3-knock down
α1AR: alpha1 adrenergic receptor
β1AR: beta1 adrenergic receptor.
